# Supplementary material for: Genomic Epidemiology Reveals the Circulation of the Chikungunya Virus East/Central/South African Lineage in Tocantins State, North Brazil
Source: Viruses. 2022 Oct 21;14(10):2311. doi: 10.3390/v14102311 (PMC9611869; doi:10.3390/v14102311)
Supplement: Supplementary file 1 [file viruses-14-02311-s001.zip › viruses-1963224-supplementary.pdf]

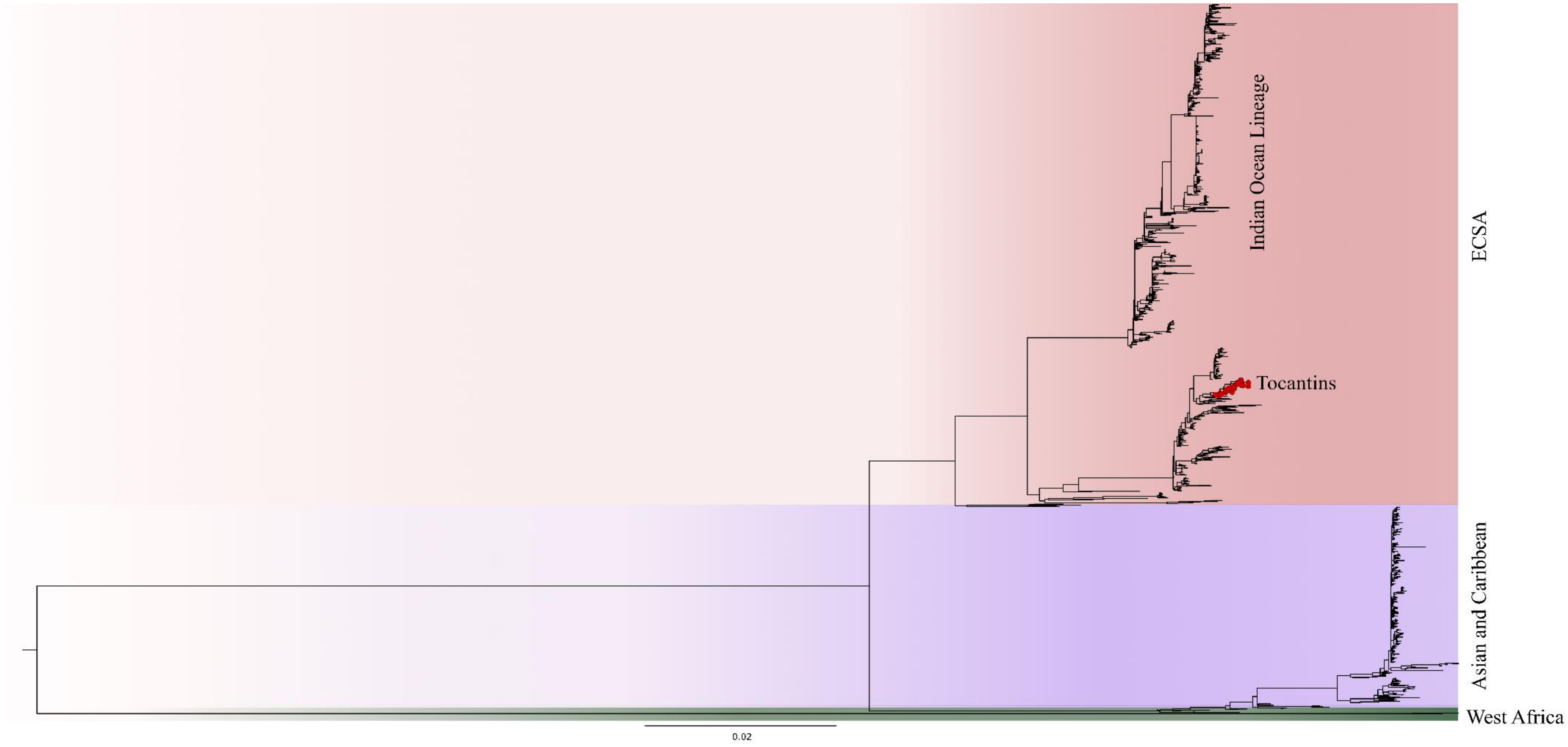

Figure S1. Maximum-likelihood tree of 1,156 genomic sequences representing the West Africa, ECSA and Asian and Caribbean genotypes.

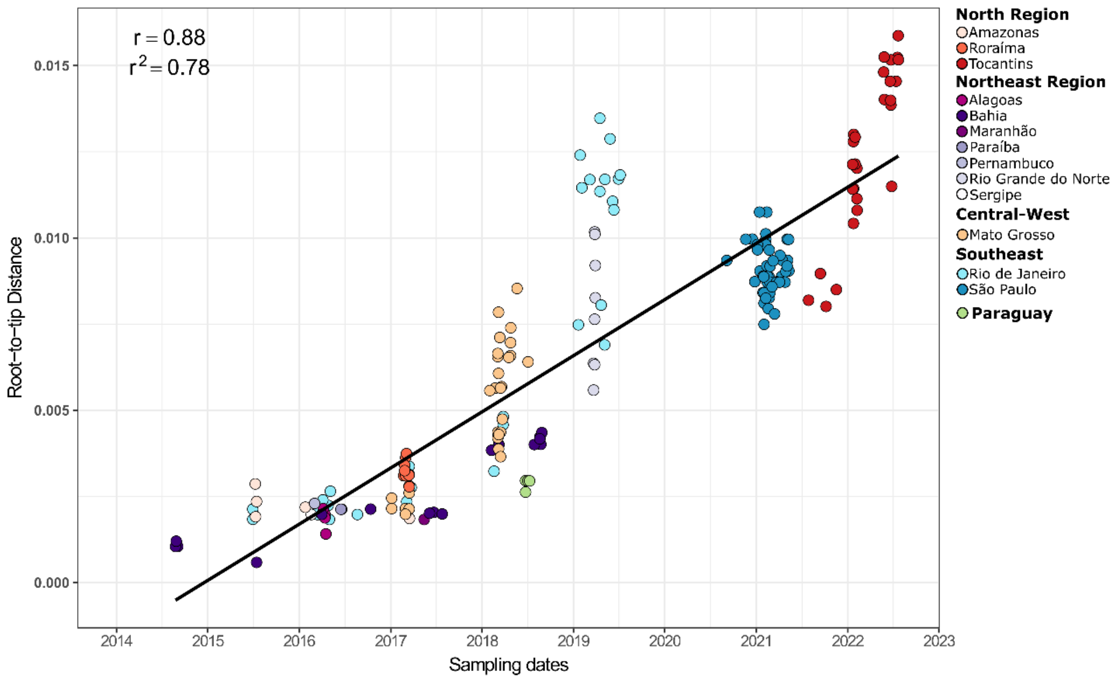

Figure S2. Root-to-tip regression of genetic distances and sampling dates for 186 Brazilian ECSA genomes in the final dataset. Correlation coefficient (r) and r squared are depicted above the graph.

Table S1. Sequences of the Chikungunya virus global dataset used in the Maximum Likelihood phylogeny.

Accession	Lineage	Date	Accession	Lineage	Date
HM045810.1	Asian and Caribbean	1905-05-11	MH647198.1	East-Central-South-African	2013-10-01
MK028840.1	Asian and Caribbean	1905-05-15	KJ579184.1	East-Central-South-African	2013-10-14
HM045788.1	Asian and Caribbean	1905-05-26	KJ579185.1	East-Central-South-African	2013-10-14
HM045814.1	Asian and Caribbean	1905-05-28	KJ579186.1	East-Central-South-African	2013-10-14
HM045808.1	Asian and Caribbean	1905-05-31	KJ579187.1	East-Central-South-African	2013-10-14
HM045791.1	Asian and Caribbean	1905-06-05	MH647199.1	East-Central-South-African	2013-12-01
HM045797.1	Asian and Caribbean	1905-06-07	MH647200.1	East-Central-South-African	2013-12-01
HM045800.1	Asian and Caribbean	1905-06-07	MH647201.1	East-Central-South-African	2013-12-01
KX262988.1	Asian and Caribbean	1905-06-10	MH647202.1	East-Central-South-African	2014-01-01
HM045789.1	Asian and Caribbean	1905-06-10	MH647204.1	East-Central-South-African	2014-02-01
HM045787.1	Asian and Caribbean	1905-06-17	MH647203.1	East-Central-South-African	2014-02-02
HM045796.1	Asian and Caribbean	1905-06-17	MH647205.1	East-Central-South-African	2014-04-01
KX262987.1	Asian and Caribbean	1905-06-18	MT526803.1	East-Central-South-African	2014-05-23
KX168429.1	Asian and Caribbean	1905-07-01	MH647206.1	East-Central-South-African	2014-06-01
MG664851.1	Asian and Caribbean	1905-07-04	KX619422.1	East-Central-South-African	2014-07-08
KT308159.1	Asian and Caribbean	1905-07-04	KX619423.1	East-Central-South-African	2014-07-23
KT308160.1	Asian and Caribbean	1905-07-04	MT526802.1	East-Central-South-African	2014-08-20
KT308161.1	Asian and Caribbean	1905-07-04	KP164568.1	East-Central-South-African	2014-08-26
KT308162.1	Asian and Caribbean	1905-07-04	KP164569.1	East-Central-South-African	2014-08-28
KT308163.1	Asian and Caribbean	1905-07-04	KP164570.1	East-Central-South-African	2014-09-03
KC488650.1	Asian and Caribbean	1905-07-04	MW042254.1	East-Central-South-African	2014-10-01
MT228633.1	Asian and Caribbean	1905-07-05	MK121891.1	East-Central-South-African	2015-07-15
KX262991.1	Asian and Caribbean	1905-07-05	MK121892.1	East-Central-South-African	2015-07-15
AB860301.3	Asian and Caribbean	1905-07-05	MK121893.1	East-Central-South-African	2015-07-15
KY575565.1	Asian and Caribbean	1905-07-06	KU940225.1	East-Central-South-African	2015-07-15
KY575566.1	Asian and Caribbean	1905-07-06	KU940226.1	East-Central-South-African	2015-08-01
KY575569.1	Asian and Caribbean	1905-07-06	MT526801.1	East-Central-South-African	2015-09-02
KY575572.1	Asian and Caribbean	1905-07-06	MH647207.1	East-Central-South-African	2015-10-01
KY575573.1	Asian and Caribbean	1905-07-06	MT526807.1	East-Central-South-African	2016-01-26
KU365369.1	Asian and Caribbean	1905-07-06	MK121894.1	East-Central-South-African	2016-01-31
KY272961.1	Asian and Caribbean	1905-07-06	MK244635.1	East-Central-South-African	2016-02-19
KY272962.1	Asian and Caribbean	1905-07-06	KY055011.1	East-Central-South-African	2016-02-20
KY272963.1	Asian and Caribbean	1905-07-06	KX228391.1	East-Central-South-African	2016-03-03
KY272964.1	Asian and Caribbean	1905-07-06	KY124328.1	East-Central-South-African	2016-03-16
KY272965.1	Asian and Caribbean	1905-07-06	KY124329.1	East-Central-South-African	2016-03-16
KY272966.1	Asian and Caribbean	1905-07-06	MG649983.1	East-Central-South-African	2016-03-28
KY272967.1	Asian and Caribbean	1905-07-06	MG649970.1	East-Central-South-African	2016-03-29
KY272968.1	Asian and Caribbean	1905-07-06	MG649976.1	East-Central-South-African	2016-03-29
KY272969.1	Asian and Caribbean	1905-07-06	MN783352.1	East-Central-South-African	2016-04-01
KY272970.1	Asian and Caribbean	1905-07-06	MK244632.1	East-Central-South-African	2016-04-05
KT581023.1	Asian and Caribbean	1905-07-06	MK244638.1	East-Central-South-African	2016-04-05
KT327164.1	Asian and Caribbean	1905-07-06	KY704952.1	East-Central-South-African	2016-04-07
KX097982.1	Asian and Caribbean	1905-07-07	MG649974.1	East-Central-South-African	2016-04-08
KX097986.1	Asian and Caribbean	1905-07-07	KY704947.1	East-Central-South-African	2016-04-15

Accession	Lineage	Date	Accession	Lineage	Date
KX097988.1	Asian and Caribbean	1905-07-07	KY704942.1	East-Central-South-African	2016-04-16
MF001507.1	Asian and Caribbean	1905-07-07	KY704939.1	East-Central-South-African	2016-04-17
MF001508.1	Asian and Caribbean	1905-07-07	MK244639.1	East-Central-South-African	2016-04-19
MF001510.1	Asian and Caribbean	1905-07-07	MK244634.1	East-Central-South-African	2016-04-27
MF001512.1	Asian and Caribbean	1905-07-07	MG649980.1	East-Central-South-African	2016-04-27
MF001513.1	Asian and Caribbean	1905-07-07	MK244636.1	East-Central-South-African	2016-05-02
KU355832.1	Asian and Caribbean	1905-07-07	MG649972.1	East-Central-South-African	2016-05-05
HM045803.1	Asian and Caribbean	1963-11-06	MK244633.1	East-Central-South-African	2016-05-06
HM045813.1	Asian and Caribbean	1963-11-06	MH423797.1	East-Central-South-African	2016-05-07
HM045790.1	Asian and Caribbean	1985-07-17	MH423798.1	East-Central-South-African	2016-05-08
FN295483.3	Asian and Caribbean	2006-03-01	MK244637.1	East-Central-South-African	2016-05-10
FN295484.2	Asian and Caribbean	2006-03-01	LC259094.1	East-Central-South-African	2016-05-11
LC259083.1	Asian and Caribbean	2009-09-11	MG000876.1	East-Central-South-African	2016-05-17
MH670649.1	Asian and Caribbean	2009-11-19	MH423799.1	East-Central-South-African	2016-05-31
HE806461.1	Asian and Caribbean	2011-02-28	MH423800.1	East-Central-South-African	2016-05-31
KY435454.1	Asian and Caribbean	2011-11-01	MH423801.1	East-Central-South-African	2016-05-31
MH647183.1	Asian and Caribbean	2012-04-01	MH423802.1	East-Central-South-African	2016-05-31
LC259084.1	Asian and Caribbean	2012-09-25	MH423803.1	East-Central-South-African	2016-05-31
LC259085.1	Asian and Caribbean	2012-10-15	MH423810.1	East-Central-South-African	2016-05-31
MH647185.1	Asian and Caribbean	2012-11-01	MH423804.1	East-Central-South-African	2016-06-01
KY883764.1	Asian and Caribbean	2013-01-01	MH423805.1	East-Central-South-African	2016-06-01
KM673291.1	Asian and Caribbean	2013-01-01	MH423806.1	East-Central-South-African	2016-06-01
MH647193.1	Asian and Caribbean	2013-05-01	KY704955.1	East-Central-South-African	2016-06-17
LC259087.1	Asian and Caribbean	2013-07-06	KY704954.1	East-Central-South-African	2016-06-20
KF872195.1	Asian and Caribbean	2013-09-24	MG000875.1	East-Central-South-African	2016-06-27
KX262992.1	Asian and Caribbean	2014-01-05	MG137428.1	East-Central-South-African	2016-06-29
KX262994.1	Asian and Caribbean	2014-01-21	MG649975.1	East-Central-South-African	2016-07-27
KY435486.1	Asian and Caribbean	2014-01-23	MG649977.1	East-Central-South-African	2016-07-28
KY435485.1	Asian and Caribbean	2014-01-28	MG649981.1	East-Central-South-African	2016-07-28
KY435484.1	Asian and Caribbean	2014-01-30	MG649971.1	East-Central-South-African	2016-08-22
KY435483.1	Asian and Caribbean	2014-02-12	MK473625.1	East-Central-South-African	2016-08-26
KY435482.1	Asian and Caribbean	2014-03-11	MF503628.1	East-Central-South-African	2016-08-26
KU365368.1	Asian and Caribbean	2014-03-26	KY057363.1	East-Central-South-African	2016-08-28
KY435481.1	Asian and Caribbean	2014-04-22	MK473621.1	East-Central-South-African	2016-09-02
KY435480.1	Asian and Caribbean	2014-04-27	MK473635.1	East-Central-South-African	2016-09-02
KY435479.1	Asian and Caribbean	2014-04-28	MK473631.1	East-Central-South-African	2016-09-11
KY680390.1	Asian and Caribbean	2014-05-07	MK473622.1	East-Central-South-African	2016-09-13
KY680354.1	Asian and Caribbean	2014-05-08	MF499120.1	East-Central-South-African	2016-09-15
KY680347.1	Asian and Caribbean	2014-05-13	MK473632.1	East-Central-South-African	2016-09-17
KY680352.1	Asian and Caribbean	2014-05-15	MK473623.1	East-Central-South-African	2016-09-20
KY680364.1	Asian and Caribbean	2014-05-15	MK473636.1	East-Central-South-African	2016-09-20
KY435478.1	Asian and Caribbean	2014-05-17	MK473637.1	East-Central-South-African	2016-09-20
KY435474.1	Asian and Caribbean	2014-05-19	MK473638.1	East-Central-South-African	2016-09-20
KY435473.1	Asian and Caribbean	2014-05-22	MK518340.1	East-Central-South-African	2016-09-28
KY435475.1	Asian and Caribbean	2014-05-22	MK551552.1	East-Central-South-African	2016-09-30
KY680361.1	Asian and Caribbean	2014-05-26	MK159124.1	East-Central-South-African	2016-10-03
KP851710.1	Asian and Caribbean	2014-05-30	MK551553.1	East-Central-South-African	2016-10-03

Accession	Lineage	Date	Accession	Lineage	Date
KY435477.1	Asian and Caribbean	2014-05-31	MK473626.1	East-Central-South-African	2016-10-06
KY680375.1	Asian and Caribbean	2014-06-04	MK159127.1	East-Central-South-African	2016-10-10
KY435476.1	Asian and Caribbean	2014-06-05	MN783353.1	East-Central-South-African	2016-10-13
KY435471.1	Asian and Caribbean	2014-06-11	MK473628.1	East-Central-South-African	2016-10-13
KY680363.1	Asian and Caribbean	2014-06-15	MK473629.1	East-Central-South-African	2016-10-18
KY435472.1	Asian and Caribbean	2014-06-16	MK473640.1	East-Central-South-African	2016-10-18
KY680351.1	Asian and Caribbean	2014-06-24	MK473633.1	East-Central-South-African	2016-10-19
KY680384.1	Asian and Caribbean	2014-06-25	MT666072.1	East-Central-South-African	2016-10-20
KY680408.1	Asian and Caribbean	2014-06-26	MK473624.1	East-Central-South-African	2016-10-23
KY680349.1	Asian and Caribbean	2014-07-02	MK473627.1	East-Central-South-African	2016-10-23
KY435460.1	Asian and Caribbean	2014-07-06	MK473639.1	East-Central-South-African	2016-10-23
KY435470.1	Asian and Caribbean	2014-07-08	MG649973.1	East-Central-South-African	2016-12-12
KY680400.1	Asian and Caribbean	2014-07-15	MH823663.1	East-Central-South-African	2017-01-05
KR264950.1	Asian and Caribbean	2014-07-16	MH823668.1	East-Central-South-African	2017-01-05
KY680395.1	Asian and Caribbean	2014-07-21	MK121897.1	East-Central-South-African	2017-02-20
KY680355.1	Asian and Caribbean	2014-07-22	MK121898.1	East-Central-South-African	2017-02-22
KY435467.1	Asian and Caribbean	2014-07-24	MK121906.1	East-Central-South-African	2017-02-27
KY435469.1	Asian and Caribbean	2014-07-30	MK121907.1	East-Central-South-African	2017-02-27
KY435463.1	Asian and Caribbean	2014-08-02	MH823666.1	East-Central-South-African	2017-03-01
KY680360.1	Asian and Caribbean	2014-08-05	MH823667.1	East-Central-South-African	2017-03-01
KY680398.1	Asian and Caribbean	2014-08-05	MK121904.1	East-Central-South-African	2017-03-02
KY435466.1	Asian and Caribbean	2014-08-06	MK121905.1	East-Central-South-African	2017-03-02
KY435468.1	Asian and Caribbean	2014-08-06	MK121896.1	East-Central-South-African	2017-03-03
KY680406.1	Asian and Caribbean	2014-08-08	MK121908.1	East-Central-South-African	2017-03-05
KY680357.1	Asian and Caribbean	2014-08-09	MK244640.1	East-Central-South-African	2017-03-07
KY435464.1	Asian and Caribbean	2014-08-15	MK244641.1	East-Central-South-African	2017-03-09
KY680377.1	Asian and Caribbean	2014-08-16	MK121903.1	East-Central-South-African	2017-03-15
KY435456.1	Asian and Caribbean	2014-08-17	MH823664.1	East-Central-South-African	2017-03-16
KY435465.1	Asian and Caribbean	2014-08-17	MH823665.1	East-Central-South-African	2017-03-16
KY680371.1	Asian and Caribbean	2014-08-20	MG649978.1	East-Central-South-African	2017-03-16
KY680410.1	Asian and Caribbean	2014-08-22	MK121899.1	East-Central-South-African	2017-03-17
KY435461.1	Asian and Caribbean	2014-08-24	MK121900.1	East-Central-South-African	2017-03-17
KY435462.1	Asian and Caribbean	2014-08-25	MK121901.1	East-Central-South-African	2017-03-17
KY680353.1	Asian and Caribbean	2014-08-25	MK121902.1	East-Central-South-African	2017-03-17
KY680412.1	Asian and Caribbean	2014-08-28	MK121895.1	East-Central-South-African	2017-03-20
KP164567.1	Asian and Caribbean	2014-08-28	MG649982.1	East-Central-South-African	2017-03-24
KY680396.1	Asian and Caribbean	2014-09-02	MT526806.1	East-Central-South-African	2017-04-03
KY680399.1	Asian and Caribbean	2014-09-05	MH349097.1	East-Central-South-African	2017-04-30
MH329296.1	Asian and Caribbean	2014-09-13	MK518395.1	East-Central-South-African	2017-05-16
KY680414.1	Asian and Caribbean	2014-09-13	MK468611.1	East-Central-South-African	2017-05-31
KY680379.1	Asian and Caribbean	2014-09-16	MK468623.1	East-Central-South-African	2017-05-31
KY435459.1	Asian and Caribbean	2014-09-17	MK468625.1	East-Central-South-African	2017-05-31
KY680413.1	Asian and Caribbean	2014-09-17	MH647208.1	East-Central-South-African	2017-06-01
KY680365.1	Asian and Caribbean	2014-09-18	MK752955.1	East-Central-South-African	2017-06-05
KY680401.1	Asian and Caribbean	2014-09-22	MK468624.1	East-Central-South-African	2017-06-14
KY680386.1	Asian and Caribbean	2014-09-24	MK468608.1	East-Central-South-African	2017-06-17
MH329303.1	Asian and Caribbean	2014-10-01	MK468612.1	East-Central-South-African	2017-06-17

Accession	Lineage	Date	Accession	Lineage	Date
KY680388.1	Asian and Caribbean	2014-10-02	MK468626.1	East-Central-South-African	2017-06-17
KY680370.1	Asian and Caribbean	2014-10-03	MK468609.1	East-Central-South-African	2017-06-19
KY703904.1	Asian and Caribbean	2014-10-04	MK468616.1	East-Central-South-African	2017-06-19
KY680394.1	Asian and Caribbean	2014-10-05	MK468617.1	East-Central-South-African	2017-06-21
KY680376.1	Asian and Caribbean	2014-10-08	MK752954.1	East-Central-South-African	2017-06-22
KU365366.1	Asian and Caribbean	2014-10-09	MK468613.1	East-Central-South-African	2017-06-24
KY680380.1	Asian and Caribbean	2014-10-13	MK468618.1	East-Central-South-African	2017-06-29
KY680387.1	Asian and Caribbean	2014-10-14	LC580236.1	East-Central-South-African	2017-07-01
MH329294.1	Asian and Caribbean	2014-10-15	LC580237.1	East-Central-South-African	2017-07-01
KP851709.1	Asian and Caribbean	2014-10-15	LC580238.1	East-Central-South-African	2017-07-01
KY680378.1	Asian and Caribbean	2014-10-19	LC580239.1	East-Central-South-African	2017-07-01
KY680392.1	Asian and Caribbean	2014-10-19	MK468619.1	East-Central-South-African	2017-07-08
KY680381.1	Asian and Caribbean	2014-10-21	MK468614.1	East-Central-South-African	2017-07-10
KY680409.1	Asian and Caribbean	2014-10-21	MK468615.1	East-Central-South-African	2017-07-10
KY680373.1	Asian and Caribbean	2014-10-22	MK468620.1	East-Central-South-African	2017-07-11
KY680391.1	Asian and Caribbean	2014-10-24	MK468621.1	East-Central-South-African	2017-07-18
KY703940.1	Asian and Caribbean	2014-10-28	MT526805.1	East-Central-South-African	2017-07-19
KY703956.1	Asian and Caribbean	2014-10-28	MK752951.1	East-Central-South-African	2017-07-27
KY435457.1	Asian and Caribbean	2014-10-30	LC580240.1	East-Central-South-African	2017-08-01
KT192707.1	Asian and Caribbean	2014-10-31	MK120197.1	East-Central-South-African	2017-08-02
MH329293.1	Asian and Caribbean	2014-11-01	MK120198.1	East-Central-South-African	2017-08-02
MH329295.1	Asian and Caribbean	2014-11-01	MK120199.1	East-Central-South-African	2017-08-02
MT038405.1	Asian and Caribbean	2014-11-03	MK120200.1	East-Central-South-African	2017-08-09
KY435458.1	Asian and Caribbean	2014-11-03	MK752953.1	East-Central-South-African	2017-08-16
KY703954.1	Asian and Caribbean	2014-11-05	MK752950.1	East-Central-South-African	2017-08-23
MK134713.1	Asian and Caribbean	2014-11-11	MK752958.1	East-Central-South-African	2017-08-23
KY435455.1	Asian and Caribbean	2014-11-12	MH400249.1	East-Central-South-African	2017-08-23
KY703947.1	Asian and Caribbean	2014-11-19	MK752952.1	East-Central-South-African	2017-08-30
KY680407.1	Asian and Caribbean	2014-11-20	MK752956.1	East-Central-South-African	2017-08-30
KY703981.1	Asian and Caribbean	2014-11-21	MG912993.1	East-Central-South-African	2017-08-30
KY680385.1	Asian and Caribbean	2014-11-25	MK468622.1	East-Central-South-African	2017-08-31
KY703950.1	Asian and Caribbean	2014-11-25	MT123008.1	East-Central-South-African	2017-09-01
KY703984.1	Asian and Caribbean	2014-11-27	MT123009.1	East-Central-South-African	2017-09-01
KY703988.1	Asian and Caribbean	2014-11-27	MT123010.1	East-Central-South-African	2017-09-01
MT038404.1	Asian and Caribbean	2014-11-30	LC580241.1	East-Central-South-African	2017-09-01
MT591085.1	Asian and Caribbean	2014-12-01	LC580242.1	East-Central-South-African	2017-09-01
KY680368.1	Asian and Caribbean	2014-12-01	LC580243.1	East-Central-South-African	2017-09-01
KU365373.1	Asian and Caribbean	2014-12-01	LC580244.1	East-Central-South-African	2017-09-01
MK134712.1	Asian and Caribbean	2014-12-03	LC580245.1	East-Central-South-African	2017-09-01
KY703959.1	Asian and Caribbean	2014-12-03	LC580246.1	East-Central-South-African	2017-09-01
KY703989.1	Asian and Caribbean	2014-12-03	LC580247.1	East-Central-South-African	2017-09-01
KY680374.1	Asian and Caribbean	2014-12-06	MK468627.1	East-Central-South-African	2017-09-18
KY703972.1	Asian and Caribbean	2014-12-09	MK120195.1	East-Central-South-African	2017-09-27
KY680350.1	Asian and Caribbean	2014-12-10	MK120196.1	East-Central-South-African	2017-09-30
KY703983.1	Asian and Caribbean	2014-12-11	LC580248.1	East-Central-South-African	2017-10-01
KY703896.1	Asian and Caribbean	2014-12-18	LC580249.1	East-Central-South-African	2017-10-01
KY703949.1	Asian and Caribbean	2015-01-15	LC580250.1	East-Central-South-African	2017-10-01

Accession	Lineage	Date	Accession	Lineage	Date
KY704002.1	Asian and Caribbean	2015-01-15	LC580251.1	East-Central-South-African	2017-10-01
KY703897.1	Asian and Caribbean	2015-01-16	LC580252.1	East-Central-South-African	2017-10-01
KY703999.1	Asian and Caribbean	2015-01-19	LC580253.1	East-Central-South-African	2017-11-01
KY704001.1	Asian and Caribbean	2015-01-21	LC580254.1	East-Central-South-African	2017-11-01
KY703968.1	Asian and Caribbean	2015-01-23	MK468610.1	East-Central-South-African	2017-11-22
KY703901.1	Asian and Caribbean	2015-01-26	LC580255.1	East-Central-South-African	2017-12-01
KY703918.1	Asian and Caribbean	2015-01-26	MT380161.1	East-Central-South-African	2017-12-01
KY703909.1	Asian and Caribbean	2015-01-28	MT526804.1	East-Central-South-African	2017-12-05
KY703919.1	Asian and Caribbean	2015-01-29	MT526800.1	East-Central-South-African	2017-12-13
KY703928.1	Asian and Caribbean	2015-01-30	MG925665.1	East-Central-South-African	2017-12-15
MT591083.1	Asian and Caribbean	2015-02-01	MT380148.1	East-Central-South-African	2018-01-01
MT591084.1	Asian and Caribbean	2015-02-01	MT380149.1	East-Central-South-African	2018-01-01
MN462649.1	Asian and Caribbean	2015-02-06	MT380150.1	East-Central-South-African	2018-01-01
MN462654.1	Asian and Caribbean	2015-02-07	MT380151.1	East-Central-South-African	2018-01-01
KY703938.1	Asian and Caribbean	2015-02-18	MT380152.1	East-Central-South-African	2018-01-01
MT150092.1	Asian and Caribbean	2015-02-24	MT380153.1	East-Central-South-African	2018-01-01
MT150093.1	Asian and Caribbean	2015-03-01	MT526799.1	East-Central-South-African	2018-01-03
MT150094.1	Asian and Caribbean	2015-03-01	MT526798.1	East-Central-South-African	2018-01-12
MN462638.1	Asian and Caribbean	2015-03-24	MT526796.1	East-Central-South-African	2018-01-15
MN462640.1	Asian and Caribbean	2015-04-05	MT380146.1	East-Central-South-African	2018-02-01
MN462658.1	Asian and Caribbean	2015-04-08	MT380147.1	East-Central-South-African	2018-02-01
MT038406.1	Asian and Caribbean	2015-04-26	MN428506.1	East-Central-South-African	2018-02-01
MT150095.1	Asian and Caribbean	2015-04-26	MK244642.1	East-Central-South-African	2018-02-18
MT150100.1	Asian and Caribbean	2015-04-27	MN428504.1	East-Central-South-African	2018-02-23
MT038407.1	Asian and Caribbean	2015-04-28	MN428524.1	East-Central-South-African	2018-03-06
MT038408.1	Asian and Caribbean	2015-05-03	MN428525.1	East-Central-South-African	2018-03-06
MT150096.1	Asian and Caribbean	2015-05-04	MN428526.1	East-Central-South-African	2018-03-06
MT150097.1	Asian and Caribbean	2015-05-04	MN428527.1	East-Central-South-African	2018-03-06
MT150098.1	Asian and Caribbean	2015-05-04	MN428518.1	East-Central-South-African	2018-03-08
MT150099.1	Asian and Caribbean	2015-05-04	MN428522.1	East-Central-South-African	2018-03-08
MN462641.1	Asian and Caribbean	2015-05-05	MN428523.1	East-Central-South-African	2018-03-08
MT038409.1	Asian and Caribbean	2015-05-06	MN428520.1	East-Central-South-African	2018-03-09
LC259091.1	Asian and Caribbean	2015-05-07	MN428509.1	East-Central-South-African	2018-03-13
MN462644.1	Asian and Caribbean	2015-05-15	MN428512.1	East-Central-South-African	2018-03-13
MN462645.1	Asian and Caribbean	2015-05-26	MN428514.1	East-Central-South-African	2018-03-13
MN462646.1	Asian and Caribbean	2015-05-27	MN428519.1	East-Central-South-African	2018-03-16
MN462647.1	Asian and Caribbean	2015-05-28	MN428521.1	East-Central-South-African	2018-03-16
MN462648.1	Asian and Caribbean	2015-05-28	MN428517.1	East-Central-South-African	2018-03-17
MN462650.1	Asian and Caribbean	2015-06-16	MN428515.1	East-Central-South-African	2018-03-20
MN462651.1	Asian and Caribbean	2015-06-24	MN428516.1	East-Central-South-African	2018-03-23
MN462652.1	Asian and Caribbean	2015-06-30	MK244644.1	East-Central-South-African	2018-03-26
MN462653.1	Asian and Caribbean	2015-06-30	MK244647.1	East-Central-South-African	2018-03-27
MN462655.1	Asian and Caribbean	2015-07-14	MK244643.1	East-Central-South-African	2018-03-28
KY680389.1	Asian and Caribbean	2015-07-14	MK244645.1	East-Central-South-African	2018-03-28
KY703907.1	Asian and Caribbean	2015-07-15	MK244646.1	East-Central-South-African	2018-03-28
MN462656.1	Asian and Caribbean	2015-07-23	MT380154.1	East-Central-South-African	2018-04-01
KY680366.1	Asian and Caribbean	2015-07-24	MT380155.1	East-Central-South-African	2018-04-01

Accession	Lineage	Date	Accession	Lineage	Date
MN462657.1	Asian and Caribbean	2015-07-29	MT380156.1	East-Central-South-African	2018-04-01
KY703915.1	Asian and Caribbean	2015-08-03	MT380160.1	East-Central-South-African	2018-04-01
KY703977.1	Asian and Caribbean	2015-08-03	MT380162.1	East-Central-South-African	2018-04-01
MN462639.1	Asian and Caribbean	2015-08-04	MK244649.1	East-Central-South-African	2018-04-03
KY703916.1	Asian and Caribbean	2015-08-04	MK244650.1	East-Central-South-African	2018-04-03
KY703942.1	Asian and Caribbean	2015-08-04	MK244648.1	East-Central-South-African	2018-04-04
KY703889.1	Asian and Caribbean	2015-08-05	MK244651.1	East-Central-South-African	2018-04-05
KY703951.1	Asian and Caribbean	2015-08-05	MK244652.1	East-Central-South-African	2018-04-05
KY703992.1	Asian and Caribbean	2015-08-05	MK244653.1	East-Central-South-African	2018-04-06
KY703961.1	Asian and Caribbean	2015-08-06	MK244654.1	East-Central-South-African	2018-04-06
KY703998.1	Asian and Caribbean	2015-08-06	MK244655.1	East-Central-South-African	2018-04-06
KY703913.1	Asian and Caribbean	2015-08-13	MK244656.1	East-Central-South-African	2018-04-06
KY703924.1	Asian and Caribbean	2015-08-13	MN428511.1	East-Central-South-African	2018-04-18
KY703995.1	Asian and Caribbean	2015-08-13	MN428510.1	East-Central-South-African	2018-04-25
MN462660.1	Asian and Caribbean	2015-08-14	MN428513.1	East-Central-South-African	2018-04-26
MN462661.1	Asian and Caribbean	2015-08-14	MN428508.1	East-Central-South-African	2018-04-27
KY703888.1	Asian and Caribbean	2015-08-15	MT380157.1	East-Central-South-African	2018-05-01
KY703914.1	Asian and Caribbean	2015-08-15	MT380158.1	East-Central-South-African	2018-05-01
KY703948.1	Asian and Caribbean	2015-08-18	MT380159.1	East-Central-South-African	2018-05-01
KY703932.1	Asian and Caribbean	2015-08-19	MT526797.1	East-Central-South-African	2018-05-15
KY703926.1	Asian and Caribbean	2015-08-20	MN428505.1	East-Central-South-African	2018-05-21
KY703898.1	Asian and Caribbean	2015-08-25	MN974214.1	East-Central-South-African	2018-06-20
MN462662.1	Asian and Caribbean	2015-08-27	MN974215.1	East-Central-South-African	2018-06-23
KY703893.1	Asian and Caribbean	2015-09-02	MT038399.1	East-Central-South-African	2018-06-25
KY703965.1	Asian and Caribbean	2015-09-03	MT038401.1	East-Central-South-African	2018-06-25
KY703974.1	Asian and Caribbean	2015-09-03	MK468801.1	East-Central-South-African	2018-06-27
KY703902.1	Asian and Caribbean	2015-09-05	MT666073.1	East-Central-South-African	2018-06-28
KY703903.1	Asian and Caribbean	2015-09-05	MT038400.1	East-Central-South-African	2018-06-29
KY703957.1	Asian and Caribbean	2015-09-05	MN974216.1	East-Central-South-African	2018-06-30
KY703976.1	Asian and Caribbean	2015-09-05	MN974217.1	East-Central-South-African	2018-07-02
KY703922.1	Asian and Caribbean	2015-09-06	MN974218.1	East-Central-South-African	2018-07-02
KY703925.1	Asian and Caribbean	2015-09-06	MT038402.1	East-Central-South-African	2018-07-03
KY703980.1	Asian and Caribbean	2015-09-06	MN428507.1	East-Central-South-African	2018-07-05
KY703963.1	Asian and Caribbean	2015-09-07	MT038403.1	East-Central-South-African	2018-07-10
KY703906.1	Asian and Caribbean	2015-09-08	MN974219.1	East-Central-South-African	2018-07-13
KY703891.1	Asian and Caribbean	2015-09-17	MN974220.1	East-Central-South-African	2018-07-19
KY703905.1	Asian and Caribbean	2015-09-17	MN974221.1	East-Central-South-African	2018-07-19
KY703923.1	Asian and Caribbean	2015-09-19	MN974222.1	East-Central-South-African	2018-07-21
KY703931.1	Asian and Caribbean	2015-09-19	MN974223.1	East-Central-South-African	2018-07-21
KY703975.1	Asian and Caribbean	2015-09-22	MK040571.1	East-Central-South-African	2018-08-21
KY703979.1	Asian and Caribbean	2015-09-22	MK040570.1	East-Central-South-African	2018-08-24
KY703890.1	Asian and Caribbean	2015-09-23	MT666071.1	East-Central-South-African	2018-09-17
KY703962.1	Asian and Caribbean	2015-10-01	MK156053.1	East-Central-South-African	2018-09-21
KY703945.1	Asian and Caribbean	2015-10-02	MK156054.1	East-Central-South-African	2018-09-21
KY703944.1	Asian and Caribbean	2015-10-03	MK156055.1	East-Central-South-African	2018-09-21
KY703953.1	Asian and Caribbean	2015-10-06	MK156056.1	East-Central-South-African	2018-09-21
KY703936.1	Asian and Caribbean	2015-10-07	MK156058.1	East-Central-South-African	2018-09-21

Accession	Lineage	Date	Accession	Lineage	Date
KY703894.1	Asian and Caribbean	2015-10-09	MK156059.1	East-Central-South-African	2018-09-21
KY703933.1	Asian and Caribbean	2015-10-09	MK156060.1	East-Central-South-African	2018-09-26
KY703991.1	Asian and Caribbean	2015-10-09	MK156061.1	East-Central-South-African	2018-09-26
KY703943.1	Asian and Caribbean	2015-10-10	MK156062.1	East-Central-South-African	2018-09-26
KY703937.1	Asian and Caribbean	2015-10-15	MK156063.1	East-Central-South-African	2018-09-26
KY703964.1	Asian and Caribbean	2015-10-16	MK156064.1	East-Central-South-African	2018-09-26
KY703910.1	Asian and Caribbean	2015-10-18	MW161364.1	East-Central-South-African	2018-10-01
KY703899.1	Asian and Caribbean	2015-10-20	MW161365.1	East-Central-South-African	2018-10-01
KY703934.1	Asian and Caribbean	2015-10-27	MW161366.1	East-Central-South-African	2018-10-01
KY703941.1	Asian and Caribbean	2015-10-28	MW161367.1	East-Central-South-African	2018-10-01
KY703921.1	Asian and Caribbean	2015-11-04	MW161368.1	East-Central-South-African	2018-10-01
KY703987.1	Asian and Caribbean	2015-11-04	MW161369.1	East-Central-South-African	2018-10-01
MN462642.1	Asian and Caribbean	2015-11-05	MW161370.1	East-Central-South-African	2018-10-01
MN462643.1	Asian and Caribbean	2015-11-05	MW161371.1	East-Central-South-African	2018-10-01
KY703973.1	Asian and Caribbean	2015-11-06	MW161372.1	East-Central-South-African	2018-10-01
KY703920.1	Asian and Caribbean	2015-11-16	MW161373.1	East-Central-South-African	2018-10-01
KY703930.1	Asian and Caribbean	2015-11-24	MW161374.1	East-Central-South-African	2018-10-01
KY703958.1	Asian and Caribbean	2015-11-25	MW161375.1	East-Central-South-African	2018-10-01
KY703892.1	Asian and Caribbean	2015-11-26	MW161376.1	East-Central-South-African	2018-10-01
KY703971.1	Asian and Caribbean	2015-11-28	MW161377.1	East-Central-South-African	2018-10-01
KY703982.1	Asian and Caribbean	2015-11-30	MW161378.1	East-Central-South-African	2018-10-01
MT591092.1	Asian and Caribbean	2015-12-01	MW161379.1	East-Central-South-African	2018-10-01
MT591093.1	Asian and Caribbean	2015-12-01	MW161380.1	East-Central-South-African	2018-10-01
MT591094.1	Asian and Caribbean	2015-12-01	MW161381.1	East-Central-South-African	2018-10-01
MT591095.1	Asian and Caribbean	2015-12-01	MW161382.1	East-Central-South-African	2018-10-01
MT591096.1	Asian and Caribbean	2015-12-01	MW161383.1	East-Central-South-African	2018-10-01
MT591097.1	Asian and Caribbean	2015-12-01	MW161384.1	East-Central-South-African	2018-10-01
MT591098.1	Asian and Caribbean	2015-12-01	MW161385.1	East-Central-South-African	2018-10-01
MT591099.1	Asian and Caribbean	2015-12-01	MW161386.1	East-Central-South-African	2018-10-01
MT591100.1	Asian and Caribbean	2015-12-01	MW161387.1	East-Central-South-African	2018-10-01
MT591101.1	Asian and Caribbean	2015-12-01	MW161388.1	East-Central-South-African	2018-10-01
MT591102.1	Asian and Caribbean	2015-12-01	MW161389.1	East-Central-South-African	2018-10-01
MT591103.1	Asian and Caribbean	2015-12-01	MW161390.1	East-Central-South-African	2018-10-01
KY703939.1	Asian and Caribbean	2015-12-01	MW161391.1	East-Central-South-African	2018-10-01
KY703929.1	Asian and Caribbean	2015-12-02	MW161392.1	East-Central-South-African	2018-10-01
KY703935.1	Asian and Caribbean	2015-12-02	MW161393.1	East-Central-South-African	2018-10-01
MN462659.1	Asian and Caribbean	2015-12-08	MW161394.1	East-Central-South-African	2018-10-01
KY703966.1	Asian and Caribbean	2015-12-10	MW161395.1	East-Central-South-African	2018-10-01
KY703900.1	Asian and Caribbean	2015-12-14	MW161396.1	East-Central-South-African	2018-10-01
KY703912.1	Asian and Caribbean	2015-12-14	MW161397.1	East-Central-South-African	2018-10-01
KY703911.1	Asian and Caribbean	2015-12-17	MW161398.1	East-Central-South-African	2018-10-01
KY703997.1	Asian and Caribbean	2015-12-19	MW161399.1	East-Central-South-African	2018-10-01
KY703996.1	Asian and Caribbean	2015-12-26	MW161400.1	East-Central-South-African	2018-10-01
MT591086.1	Asian and Caribbean	2016-01-01	MW161401.1	East-Central-South-African	2018-10-01
MT591087.1	Asian and Caribbean	2016-01-01	MW161402.1	East-Central-South-African	2018-10-01
MT591088.1	Asian and Caribbean	2016-01-01	MW161403.1	East-Central-South-African	2018-10-01
MT591089.1	Asian and Caribbean	2016-01-01	MW161404.1	East-Central-South-African	2018-10-01

Accession	Lineage	Date	Accession	Lineage	Date
KY703917.1	Asian and Caribbean	2016-01-08	MW161405.1	East-Central-South-African	2018-10-01
MT038393.1	Asian and Caribbean	2016-01-20	MW161406.1	East-Central-South-African	2018-10-01
MT038397.1	Asian and Caribbean	2016-01-21	MW161407.1	East-Central-South-African	2018-10-01
MT038395.1	Asian and Caribbean	2016-02-16	MW161408.1	East-Central-South-African	2018-10-01
MT038394.1	Asian and Caribbean	2016-02-19	MW161409.1	East-Central-South-African	2018-10-01
MT038396.1	Asian and Caribbean	2016-02-22	MW161410.1	East-Central-South-African	2018-10-01
MT038398.1	Asian and Caribbean	2016-03-21	MW161411.1	East-Central-South-African	2018-10-01
OK655884.1	Asian and Caribbean	2016-11-21	MW161412.1	East-Central-South-African	2018-10-01
OK655883.1	Asian and Caribbean	2016-11-24	MW161413.1	East-Central-South-African	2018-10-01
MT591104.1	Asian and Caribbean	2017-01-01	MW161414.1	East-Central-South-African	2018-10-01
MT591090.1	Asian and Caribbean	2017-02-01	MW161415.1	East-Central-South-African	2018-10-01
MT591091.1	Asian and Caribbean	2017-03-01	MW161416.1	East-Central-South-African	2018-10-01
MT591105.1	Asian and Caribbean	2018-03-01	MW161417.1	East-Central-South-African	2018-10-01
MT591106.1	Asian and Caribbean	2018-05-01	MW161418.1	East-Central-South-African	2018-10-01
MT591107.1	Asian and Caribbean	2018-07-01	MW161419.1	East-Central-South-African	2018-10-01
HM045809.1	East-Central-South-African	1905-05-13	MW161420.1	East-Central-South-African	2018-10-01
HM045795.1	East-Central-South-African	1905-05-29	MW161421.1	East-Central-South-African	2018-10-01
HM045812.1	East-Central-South-African	1905-06-04	MW161422.1	East-Central-South-African	2018-10-01
KY575574.1	East-Central-South-African	1905-06-17	MW161423.1	East-Central-South-African	2018-10-01
KP702297.1	East-Central-South-African	1905-06-27	MW161424.1	East-Central-South-African	2018-10-01
KF283986.1	East-Central-South-African	1905-06-27	MW161425.1	East-Central-South-African	2018-10-01
KF283987.1	East-Central-South-African	1905-06-27	MW161426.1	East-Central-South-African	2018-10-01
MK028838.1	East-Central-South-African	1905-06-28	MW161427.1	East-Central-South-African	2018-10-01
KY575567.1	East-Central-South-African	1905-06-28	MW161428.1	East-Central-South-African	2018-10-01
KY575568.1	East-Central-South-African	1905-06-28	MW161429.1	East-Central-South-African	2018-10-01
KY575571.1	East-Central-South-African	1905-06-28	MW161430.1	East-Central-South-African	2018-10-01
KX262996.1	East-Central-South-African	1905-06-28	MW161431.1	East-Central-South-African	2018-10-01
KP003807.1	East-Central-South-African	1905-06-28	MW161432.1	East-Central-South-African	2018-10-01
KP003808.1	East-Central-South-African	1905-06-28	MW161433.1	East-Central-South-African	2018-10-01
KP003809.1	East-Central-South-African	1905-06-28	MW161434.1	East-Central-South-African	2018-10-01
KJ941050.1	East-Central-South-African	1905-06-28	MW161435.1	East-Central-South-African	2018-10-01
HM045794.1	East-Central-South-African	1905-06-28	MW161436.1	East-Central-South-African	2018-10-01
GU189061.1	East-Central-South-African	1905-06-28	MW161437.1	East-Central-South-African	2018-10-01
FJ807896.1	East-Central-South-African	1905-06-28	MW161438.1	East-Central-South-African	2018-10-01
FJ959103.1	East-Central-South-African	1905-06-28	MW161439.1	East-Central-South-African	2018-10-01
EF210157.2	East-Central-South-African	1905-06-28	MW161440.1	East-Central-South-African	2018-10-01
KX262989.1	East-Central-South-African	1905-06-29	MW161441.1	East-Central-South-African	2018-10-01
KX262993.1	East-Central-South-African	1905-06-29	MW161442.1	East-Central-South-African	2018-10-01
KP003810.1	East-Central-South-African	1905-06-29	MW161443.1	East-Central-South-African	2018-10-01
KP003811.1	East-Central-South-African	1905-06-29	MW161444.1	East-Central-South-African	2018-10-01
KP003812.1	East-Central-South-African	1905-06-29	MW161445.1	East-Central-South-African	2018-10-01
HM045799.1	East-Central-South-African	1905-06-29	MW161446.1	East-Central-South-African	2018-10-01
HM045801.1	East-Central-South-African	1905-06-29	MW161447.1	East-Central-South-African	2018-10-01
MF773568.1	East-Central-South-African	1905-06-30	MW161448.1	East-Central-South-African	2018-10-01
KY575570.1	East-Central-South-African	1905-06-30	MW161449.1	East-Central-South-African	2018-10-01
JN558835.1	East-Central-South-African	1905-06-30	MW161450.1	East-Central-South-African	2018-10-01
GU199350.1	East-Central-South-African	1905-06-30	MW161451.1	East-Central-South-African	2018-10-01

Accession	Lineage	Date	Accession	Lineage	Date
GU199351.1	East-Central-South-African	1905-06-30	MW161452.1	East-Central-South-African	2018-10-01
GU199352.1	East-Central-South-African	1905-06-30	MW161453.1	East-Central-South-African	2018-10-01
GU199353.1	East-Central-South-African	1905-06-30	MW161454.1	East-Central-South-African	2018-10-01
FJ807898.1	East-Central-South-African	1905-06-30	MW161455.1	East-Central-South-African	2018-10-01
FJ807899.1	East-Central-South-African	1905-06-30	MW161456.1	East-Central-South-African	2018-10-01
FN295485.3	East-Central-South-African	1905-06-30	MW161457.1	East-Central-South-African	2018-10-01
FN295487.2	East-Central-South-African	1905-06-30	MW161458.1	East-Central-South-African	2018-10-01
KX262997.1	East-Central-South-African	1905-07-01	MW161459.1	East-Central-South-African	2018-10-01
JN558834.1	East-Central-South-African	1905-07-01	MW161460.1	East-Central-South-African	2018-10-01
JN558836.1	East-Central-South-African	1905-07-01	MK163628.1	East-Central-South-African	2018-10-02
LC580270.1	East-Central-South-African	1905-07-02	MK848202.1	East-Central-South-African	2018-11-21
MH124570.1	East-Central-South-African	1905-07-02	MN974211.1	East-Central-South-African	2018-12-12
MH124571.1	East-Central-South-African	1905-07-02	MT636907.1	East-Central-South-African	2019-01-07
MH124572.1	East-Central-South-African	1905-07-02	MT636908.1	East-Central-South-African	2019-01-07
MH124573.1	East-Central-South-African	1905-07-02	MT933029.1	East-Central-South-African	2019-01-21
MH124574.1	East-Central-South-African	1905-07-02	MT636909.1	East-Central-South-African	2019-01-21
MH124575.1	East-Central-South-African	1905-07-02	MT636910.1	East-Central-South-African	2019-01-21
MH124576.1	East-Central-South-African	1905-07-02	MT636911.1	East-Central-South-African	2019-01-21
MH124577.1	East-Central-South-African	1905-07-02	MT933030.1	East-Central-South-African	2019-01-28
MH124578.1	East-Central-South-African	1905-07-02	MT636912.1	East-Central-South-African	2019-02-01
MH124579.1	East-Central-South-African	1905-07-02	MN075149.1	East-Central-South-African	2019-02-01
MG664850.1	East-Central-South-African	1905-07-02	MN075150.1	East-Central-South-African	2019-02-01
KF590564.1	East-Central-South-African	1905-07-02	MT933032.1	East-Central-South-African	2019-02-05
KF590565.1	East-Central-South-African	1905-07-02	MT636913.1	East-Central-South-African	2019-02-06
KF590566.1	East-Central-South-African	1905-07-02	MN974208.1	East-Central-South-African	2019-02-12
KC862329.1	East-Central-South-African	1905-07-02	MT933031.1	East-Central-South-African	2019-02-18
JX088705.1	East-Central-South-African	1905-07-02	MK935344.1	East-Central-South-African	2019-03-05
MF773567.1	East-Central-South-African	1905-07-03	MT933033.1	East-Central-South-African	2019-03-08
KP003813.2	East-Central-South-African	1905-07-03	MN974207.1	East-Central-South-African	2019-03-12
MF773569.1	East-Central-South-African	1905-07-05	MT933034.1	East-Central-South-African	2019-03-15
KX009167.1	East-Central-South-African	1905-07-05	MK935343.1	East-Central-South-African	2019-03-20
KX009168.1	East-Central-South-African	1905-07-05	MW260512.1	East-Central-South-African	2019-03-23
KX009169.1	East-Central-South-African	1905-07-05	MW260513.1	East-Central-South-African	2019-03-23
KX009170.1	East-Central-South-African	1905-07-05	MW260515.1	East-Central-South-African	2019-03-27
KX009171.1	East-Central-South-African	1905-07-05	MW260514.1	East-Central-South-African	2019-03-28
MK286895.1	East-Central-South-African	1905-07-06	MW260516.1	East-Central-South-African	2019-03-28
MK370030.1	East-Central-South-African	1905-07-07	MW260517.1	East-Central-South-African	2019-03-29
MK370031.1	East-Central-South-African	1905-07-07	LC664141.1	East-Central-South-African	2019-03-30
MG649984.1	East-Central-South-African	2015	LC664143.1	East-Central-South-African	2019-03-30
MG649985.1	East-Central-South-African	2015	MW260518.1	East-Central-South-African	2019-03-30
MW574902.1	East-Central-South-African	1905-07-08	MW260519.1	East-Central-South-African	2019-03-30
MW321606.1	East-Central-South-African	1905-07-08	LC664142.1	East-Central-South-African	2019-03-31
MK370032.1	East-Central-South-African	1905-07-08	LC664145.1	East-Central-South-African	2019-04-01
MK370033.1	East-Central-South-African	1905-07-08	LC664149.1	East-Central-South-African	2019-04-01
MH124580.1	East-Central-South-African	1905-07-08	LC664146.1	East-Central-South-African	2019-04-03
MH124581.1	East-Central-South-African	1905-07-08	LC664148.1	East-Central-South-African	2019-04-04
MH124582.1	East-Central-South-African	1905-07-08	LC664147.1	East-Central-South-African	2019-04-07

Accession	Lineage	Date	Accession	Lineage	Date
MH124583.1	East-Central-South-African	1905-07-08	LC664150.1	East-Central-South-African	2019-04-10
MK473630.1	East-Central-South-African	1905-07-08	LC664151.1	East-Central-South-African	2019-04-14
MK473634.1	East-Central-South-African	1905-07-08	MT495605.1	East-Central-South-African	2019-04-14
LC331252.1	East-Central-South-African	1905-07-08	MT933035.1	East-Central-South-African	2019-04-17
MF774613.1	East-Central-South-African	1905-07-08	MT933036.1	East-Central-South-African	2019-04-17
MF774614.1	East-Central-South-African	1905-07-08	MT933037.1	East-Central-South-African	2019-04-18
MF774615.1	East-Central-South-African	1905-07-08	MT933038.1	East-Central-South-African	2019-04-22
MF774616.1	East-Central-South-African	1905-07-08	LC664144.1	East-Central-South-African	2019-04-24
MF774617.1	East-Central-South-African	1905-07-08	LC664153.1	East-Central-South-African	2019-05-01
MF774618.1	East-Central-South-African	1905-07-08	LC664154.1	East-Central-South-African	2019-05-01
MF774619.1	East-Central-South-African	1905-07-08	MT933039.1	East-Central-South-African	2019-05-06
KY751908.1	East-Central-South-African	1905-07-08	MT933040.1	East-Central-South-African	2019-05-06
MK286893.1	East-Central-South-African	1905-07-09	MN402883.1	East-Central-South-African	2019-05-07
MK286894.1	East-Central-South-African	1905-07-09	MT933041.1	East-Central-South-African	2019-05-08
MK286899.1	East-Central-South-African	1905-07-09	MN402885.1	East-Central-South-African	2019-05-08
MH507158.1	East-Central-South-African	1905-07-09	MT933042.1	East-Central-South-African	2019-05-11
MF740874.1	East-Central-South-African	1905-07-09	LC664152.1	East-Central-South-African	2019-05-19
MF773566.1	East-Central-South-African	1905-07-09	MT933051.1	East-Central-South-African	2019-05-28
MG049915.1	East-Central-South-African	1905-07-09	MW042255.1	East-Central-South-African	2019-06-01
MK286897.1	East-Central-South-African	1905-07-10	MT933043.1	East-Central-South-African	2019-06-07
MK286898.1	East-Central-South-African	1905-07-10	MT933044.1	East-Central-South-African	2019-06-13
MN630017.1	East-Central-South-African	1905-07-11	LC664155.1	East-Central-South-African	2019-06-18
MK690206.1	East-Central-South-African	1905-07-11	MT933045.1	East-Central-South-African	2019-06-19
HM045811.1	East-Central-South-African	1953-02-22	MN402884.1	East-Central-South-African	2019-06-28
HM045792.1	East-Central-South-African	1956-04-01	MT933046.1	East-Central-South-African	2019-07-01
HM045822.1	East-Central-South-African	1978-10-01	MT933047.1	East-Central-South-African	2019-07-02
KY038947.2	East-Central-South-African	1983-12-01	MN402886.1	East-Central-South-African	2019-07-02
FR717336.1	East-Central-South-African	2005-12-26	MT933049.1	East-Central-South-African	2019-07-07
FR717337.1	East-Central-South-African	2005-12-26	MN974212.1	East-Central-South-African	2019-07-08
EU564334.1	East-Central-South-African	2006-02-14	MN974205.1	East-Central-South-African	2019-07-24
FJ000067.1	East-Central-South-African	2006-08-01	MN402889.1	East-Central-South-African	2019-07-25
FJ000068.1	East-Central-South-African	2006-08-01	MN402890.1	East-Central-South-African	2019-07-26
FJ000062.1	East-Central-South-African	2006-09-01	MN402891.1	East-Central-South-African	2019-07-30
FJ000064.1	East-Central-South-African	2006-09-01	MT933048.1	East-Central-South-African	2019-07-31
FJ000065.1	East-Central-South-African	2006-09-01	MN402887.1	East-Central-South-African	2019-08-01
FJ000066.1	East-Central-South-African	2006-09-01	MN402888.1	East-Central-South-African	2019-08-01
JF274082.1	East-Central-South-African	2006-09-27	LC664156.1	East-Central-South-African	2019-08-02
FJ000063.1	East-Central-South-African	2006-10-01	MN402892.1	East-Central-South-African	2019-08-02
GQ428210.1	East-Central-South-African	2006-10-07	MN756625.1	East-Central-South-African	2019-08-08
GQ428211.1	East-Central-South-African	2006-10-07	MT495606.1	East-Central-South-African	2019-08-12
EU564335.1	East-Central-South-African	2006-10-31	MT933050.1	East-Central-South-African	2019-08-14
MK086029.1	East-Central-South-African	2006-11-14	MN974204.1	East-Central-South-African	2019-08-15
FJ445428.2	East-Central-South-African	2007-05-01	MN974210.1	East-Central-South-African	2019-08-26
FJ000069.1	East-Central-South-African	2007-06-01	MT495607.1	East-Central-South-African	2019-08-28
EU372006.1	East-Central-South-African	2007-06-11	MN974206.1	East-Central-South-African	2019-08-30
FJ445427.2	East-Central-South-African	2007-07-01	MN974224.1	East-Central-South-African	2019-09-06
GQ428212.1	East-Central-South-African	2007-07-12	MN974203.1	East-Central-South-African	2019-09-11

Accession	Lineage	Date	Accession	Lineage	Date
GQ428213.1	East-Central-South-African	2007-07-13	MW110472.1	East-Central-South-African	2019-09-28
MK120201.1	East-Central-South-African	2007-08-20	LC580256.1	East-Central-South-African	2019-10-01
MK120202.1	East-Central-South-African	2007-08-25	LC580257.1	East-Central-South-African	2019-10-01
FJ445510.2	East-Central-South-African	2008-01-01	LC580258.1	East-Central-South-African	2019-10-01
FJ445511.2	East-Central-South-African	2008-01-01	LC580259.1	East-Central-South-African	2019-10-01
GU013528.2	East-Central-South-African	2008-03-01	LC580260.1	East-Central-South-African	2019-10-01
GU013529.2	East-Central-South-African	2008-03-01	LC580261.1	East-Central-South-African	2019-10-01
FJ513628.1	East-Central-South-African	2008-03-01	LC580262.1	East-Central-South-African	2019-10-01
FJ513629.1	East-Central-South-African	2008-03-01	LC580263.1	East-Central-South-African	2019-10-01
FJ513632.1	East-Central-South-African	2008-03-01	LC580264.1	East-Central-South-African	2019-10-01
FJ513635.1	East-Central-South-African	2008-03-01	LC580265.1	East-Central-South-African	2019-10-01
FJ513637.1	East-Central-South-African	2008-03-01	LC580266.1	East-Central-South-African	2019-10-01
GU013530.2	East-Central-South-African	2008-04-01	LC580267.1	East-Central-South-African	2019-10-01
FJ513645.1	East-Central-South-African	2008-04-01	LC580268.1	East-Central-South-African	2019-10-01
FJ513654.1	East-Central-South-African	2008-04-01	LC580269.1	East-Central-South-African	2019-10-01
FJ513657.1	East-Central-South-African	2008-04-01	MW291576.1	East-Central-South-African	2019-10-04
FJ513673.1	East-Central-South-African	2008-04-01	MW110473.1	East-Central-South-African	2019-10-06
FJ513675.1	East-Central-South-African	2008-04-01	MW110474.1	East-Central-South-African	2019-10-06
FJ513679.1	East-Central-South-African	2008-04-01	MN974209.1	East-Central-South-African	2019-10-07
FJ445426.2	East-Central-South-African	2008-04-01	MN974213.1	East-Central-South-African	2019-10-07
FJ445484.2	East-Central-South-African	2008-05-01	MW110475.1	East-Central-South-African	2019-10-08
GQ428215.1	East-Central-South-African	2008-05-29	MW110476.1	East-Central-South-African	2019-10-12
GQ428214.1	East-Central-South-African	2008-06-29	MW110477.1	East-Central-South-African	2019-10-12
FJ445430.2	East-Central-South-African	2008-07-01	MT668625.1	East-Central-South-African	2019-11-04
FJ445431.2	East-Central-South-African	2008-07-01	MT495608.1	East-Central-South-African	2019-11-06
FJ445432.2	East-Central-South-African	2008-07-01	MT023791.1	East-Central-South-African	2019-12-15
FJ445463.2	East-Central-South-African	2008-07-01	MT640255.1	East-Central-South-African	2020-01-17
FJ445433.2	East-Central-South-African	2008-08-01	MT640256.1	East-Central-South-African	2020-02-05
FJ445443.2	East-Central-South-African	2008-08-01	LC664157.1	East-Central-South-African	2020-06-29
FJ445445.2	East-Central-South-African	2008-08-01	OL705486.1	East-Central-South-African	2020-07-15
FJ445502.2	East-Central-South-African	2008-08-01	LC664158.1	East-Central-South-African	2020-07-21
FR687340.1	East-Central-South-African	2008-08-21	OL741630.1	East-Central-South-African	2020-07-23
GU301780.1	East-Central-South-African	2008-10-21	LC664159.1	East-Central-South-African	2020-08-04
FR687341.1	East-Central-South-African	2008-11-05	LC664160.1	East-Central-South-African	2020-08-19
FR687342.1	East-Central-South-African	2008-11-10	LC664161.1	East-Central-South-African	2020-08-20
KT324224.1	East-Central-South-African	2008-12-01	LC664162.1	East-Central-South-African	2020-08-26
FR687343.1	East-Central-South-African	2008-12-12	LC664163.1	East-Central-South-African	2020-08-28
KT324225.1	East-Central-South-African	2009-01-01	OL898663.1	East-Central-South-African	2020-09-05
KT324226.1	East-Central-South-African	2009-01-01	LC664164.1	East-Central-South-African	2020-09-08
LC259093.1	East-Central-South-African	2009-01-06	LC664165.1	East-Central-South-African	2020-09-13
FR687344.1	East-Central-South-African	2009-01-12	OL849990.1	East-Central-South-African	2020-09-22
KT324228.1	East-Central-South-African	2009-02-01	LC664166.1	East-Central-South-African	2020-09-25
FR687345.1	East-Central-South-African	2009-02-05	OL849991.1	East-Central-South-African	2020-09-28
FR687347.1	East-Central-South-African	2009-02-12	OL893105.1	East-Central-South-African	2020-10-06
FR687346.1	East-Central-South-African	2009-02-16	OL893106.1	East-Central-South-African	2020-10-09
KT324227.1	East-Central-South-African	2009-03-01	OL893107.1	East-Central-South-African	2020-10-14
FR687348.1	East-Central-South-African	2009-04-03	OL979157.1	East-Central-South-African	2020-11-02

Accession	Lineage	Date	Accession	Lineage	Date
GQ905863.1	East-Central-South-African	2009-05-25	OL979156.1	East-Central-South-African	2020-11-16
KF151174.1	East-Central-South-African	2009-07-13	OL979155.1	East-Central-South-African	2020-11-18
GU301781.1	East-Central-South-African	2009-07-27	OL898714.1	East-Central-South-African	2020-11-20
GU301779.1	East-Central-South-African	2009-09-04	OL979154.1	East-Central-South-African	2020-12-07
MH647212.1	East-Central-South-African	2009-11-01	OL898669.1	East-Central-South-African	2020-12-17
MH647210.1	East-Central-South-African	2009-12-01	OL979153.1	East-Central-South-African	2020-12-24
MH647211.1	East-Central-South-African	2009-12-01	OL999091.1	East-Central-South-African	2020-12-25
KF151175.1	East-Central-South-African	2009-12-11	OL898675.1	East-Central-South-African	2020-12-26
MH636708.1	East-Central-South-African	2010-01-01	OL898668.1	East-Central-South-African	2021-01-06
MH647213.1	East-Central-South-African	2010-01-01	OL898682.1	East-Central-South-African	2021-01-07
MH647214.1	East-Central-South-African	2010-01-01	OL898707.1	East-Central-South-African	2021-01-07
MH647216.1	East-Central-South-African	2010-01-01	OL898689.1	East-Central-South-African	2021-01-15
MH647215.1	East-Central-South-African	2010-02-01	OL898690.1	East-Central-South-African	2021-01-16
JQ065885.1	East-Central-South-African	2010-10-01	OL898674.1	East-Central-South-African	2021-01-23
JQ065886.1	East-Central-South-African	2010-10-01	OL898696.1	East-Central-South-African	2021-01-28
JQ065887.1	East-Central-South-African	2010-10-01	OL898700.1	East-Central-South-African	2021-01-28
JQ065888.1	East-Central-South-African	2010-10-01	OL898704.1	East-Central-South-African	2021-01-28
JQ065889.1	East-Central-South-African	2010-10-01	OL898664.1	East-Central-South-African	2021-01-31
JQ065890.1	East-Central-South-African	2010-10-01	OL898683.1	East-Central-South-African	2021-01-31
JQ065891.1	East-Central-South-African	2010-10-01	OL898667.1	East-Central-South-African	2021-02-01
JQ065892.1	East-Central-South-African	2010-10-01	OL898670.1	East-Central-South-African	2021-02-02
HQ846356.1	East-Central-South-African	2010-10-01	OL898676.1	East-Central-South-African	2021-02-02
HQ846357.1	East-Central-South-African	2010-10-01	OL898710.1	East-Central-South-African	2021-02-02
HQ846358.1	East-Central-South-African	2010-10-01	OL898711.1	East-Central-South-African	2021-02-08
HQ846359.1	East-Central-South-African	2010-10-01	OL898697.1	East-Central-South-African	2021-02-09
JQ861259.1	East-Central-South-African	2011-05-26	OL898699.1	East-Central-South-African	2021-02-09
JQ861260.1	East-Central-South-African	2011-05-28	OL898706.1	East-Central-South-African	2021-02-09
JQ861253.1	East-Central-South-African	2011-08-16	OL999092.1	East-Central-South-African	2021-02-12
JQ861254.1	East-Central-South-African	2011-08-16	OL898677.1	East-Central-South-African	2021-02-13
JQ861255.1	East-Central-South-African	2011-08-16	OL898693.1	East-Central-South-African	2021-02-13
JQ861256.1	East-Central-South-African	2011-08-16	OL898705.1	East-Central-South-African	2021-02-13
JQ861257.1	East-Central-South-African	2011-08-16	OL898684.1	East-Central-South-African	2021-02-14
JQ861258.1	East-Central-South-African	2011-08-16	OL898671.1	East-Central-South-African	2021-02-16
KJ679577.1	East-Central-South-African	2011-09-12	OL898712.1	East-Central-South-African	2021-02-18
MH647180.1	East-Central-South-African	2011-10-01	OL898681.1	East-Central-South-African	2021-02-20
MH647181.1	East-Central-South-African	2011-11-01	OL898691.1	East-Central-South-African	2021-02-21
KU365370.1	East-Central-South-African	2011-11-01	OL898665.1	East-Central-South-African	2021-02-22
KU365371.1	East-Central-South-African	2011-11-01	OL898685.1	East-Central-South-African	2021-02-22
KJ679578.1	East-Central-South-African	2011-12-21	OL999093.1	East-Central-South-African	2021-02-24
MH647182.1	East-Central-South-African	2012-01-01	OL898666.1	East-Central-South-African	2021-02-25
MF076573.1	East-Central-South-African	2012-08-07	OL898672.1	East-Central-South-African	2021-02-25
MF076574.1	East-Central-South-African	2012-08-16	OL898698.1	East-Central-South-African	2021-02-25
MF076575.1	East-Central-South-African	2012-08-16	OL898678.1	East-Central-South-African	2021-03-05
MF076576.1	East-Central-South-African	2012-08-31	OL999094.1	East-Central-South-African	2021-03-05
MH647184.1	East-Central-South-African	2012-10-01	OL898692.1	East-Central-South-African	2021-03-09
MH647186.1	East-Central-South-African	2012-11-01	OL898673.1	East-Central-South-African	2021-03-15
MH647187.1	East-Central-South-African	2013-02-01	OL898680.1	East-Central-South-African	2021-03-22

Accession	Lineage	Date	Accession	Lineage	Date
MH647217.1	East-Central-South-African	2013-02-01	OL898688.1	East-Central-South-African	2021-03-23
MH647218.1	East-Central-South-African	2013-03-01	OL898686.1	East-Central-South-African	2021-03-25
MF076572.1	East-Central-South-African	2013-03-12	OL898695.1	East-Central-South-African	2021-04-04
MF076568.1	East-Central-South-African	2013-03-28	OL898703.1	East-Central-South-African	2021-04-06
MF076569.1	East-Central-South-African	2013-03-28	OL898679.1	East-Central-South-African	2021-04-13
MF076570.1	East-Central-South-African	2013-03-28	OL898687.1	East-Central-South-African	2021-04-16
MF076571.1	East-Central-South-African	2013-03-28	OL898702.1	East-Central-South-African	2021-04-27
MH647188.1	East-Central-South-African	2013-04-01	OL898713.1	East-Central-South-African	2021-04-29
MH647189.1	East-Central-South-African	2013-04-01	OL898701.1	East-Central-South-African	2021-05-05
MH647190.1	East-Central-South-African	2013-05-01	OL898708.1	East-Central-South-African	2021-05-05
MH647191.1	East-Central-South-African	2013-05-01	OL898715.1	East-Central-South-African	2021-05-06
MH647192.1	East-Central-South-African	2013-05-01	OL898694.1	East-Central-South-African	2021-05-11
MH647194.1	East-Central-South-African	2013-06-01	OL898709.1	East-Central-South-African	2021-05-12
MH647209.1	East-Central-South-African	2013-06-01	OL999095.1	East-Central-South-African	2021-05-24
MH647195.1	East-Central-South-African	2013-07-01	MG649979.1	East-Central-South-African	2017-03
MH647196.1	East-Central-South-African	2013-08-01	HM045786.1	West African	1964-07-07
MH647197.1	East-Central-South-African	2013-10-01	HM045798.1	West African	1966-11-01

Table S2. Full details of the 186 complete and near-complete CHIKV genome sequences from the ECSA genotype samples in Brazil and Americas used in this study.

Acession Number	State	Country	Collection date
KP164568.1	Bahia	Brazil	2014-08-26
KP164569.1	Bahia	Brazil	2014-08-28
KP164570.1	Bahia	Brazil	2014-09-03
MK121891.1	Amazonas	Brazil	2015-07-10
MK121892.1	Amazonas	Brazil	2015-07-11
MK121893.1	Amazonas	Brazil	2015-07-15
KU940225.1	Bahia	Brazil	2015-07-15
MG649984.1	Rio de Janeiro	Brazil	1905-07-07
MG649985.1	Rio de Janeiro	Brazil	1905-07-07
MK121894.1	Amazonas	Brazil	2016-01-27
KY055011.1	Sergipe	Brazil	2016-02-20
KX228391.1	Pernambuco	Brazil	2016-03-03
KY124328.1	Rio de Janeiro	Brazil	2016-03-16
KY124329.1	Rio de Janeiro	Brazil	2016-03-16
MN783352.1	Bahia	Brazil	2016-04-01
MK244638.1	Rio de Janeiro	Brazil	2016-04-05
KY704952.1	Alagoas	Brazil	2016-04-07
KY704942.1	Alagoas	Brazil	2016-04-14
KY704947.1	Alagoas	Brazil	2016-04-15
KY704939.1	Alagoas	Brazil	2016-04-17
MK244634.1	Rio de Janeiro	Brazil	2016-04-27
MK244636.1	Rio de Janeiro	Brazil	2016-05-02
MK244633.1	Rio de Janeiro	Brazil	2016-05-06
KY704955.1	Joao Pessoa	Brazil	2016-06-17
KY704954.1	Joao Pessoa	Brazil	2016-06-20
MG649971.1	Rio de Janeiro	Brazil	2016-08-22
MN783353.1	Bahia	Brazil	2016-10-13
MH823663.1	Mato Grosso	Brazil	2017-01-05
MH823668.1	Mato Grosso	Brazil	2017-01-05
MK121898.1	Roraima	Brazil	2017-02-22
MK121906.1	Roraima	Brazil	2017-02-27
MK121907.1	Roraima	Brazil	2017-02-27
MH823666.1	Mato Grosso	Brazil	2017-03-01
MH823667.1	Mato Grosso	Brazil	2017-03-01
MK121904.1	Roraima	Brazil	2017-03-02
MK121896.1	Roraima	Brazil	2017-03-03
MK121908.1	Roraima	Brazil	2017-03-05
MK244640.1	Rio de Janeiro	Brazil	2017-03-07
MK121903.1	Roraima	Brazil	2017-03-15
MH823664.1	Mato Grosso	Brazil	2017-03-16
MH823665.1	Mato Grosso	Brazil	2017-03-16
MG649978.1	Rio de Janeiro	Brazil	2017-03-16
MK121899.1	Roraima	Brazil	2017-03-17
MK121900.1	Roraima	Brazil	2017-03-17

Acession Number	State	Country	Collection date
MK121901.1	Roraima	Brazil	2017-03-17
MK121902.1	Roraima	Brazil	2017-03-17
MK121895.1	Amazonas	Brazil	2017-03-18
MG649982.1	Rio de Janeiro	Brazil	2017-03-24
MK518395.1	Maranhão	Brazil	2017-05-16
MK752955.1	Bahia	Brazil	2017-06-05
MK752954.1	Bahia	Brazil	2017-06-22
MK752951.1	Bahia	Brazil	2017-07-27
MN428506.1	Mato Grosso	Brazil	2018-02-01
MK156060.1	Bahia	Brazil	2018-02-08
MK244642.1	Rio de Janeiro	Brazil	2018-02-18
MN428504.1	Mato Grosso	Brazil	2018-02-23
MN428524.1	Mato Grosso	Brazil	2018-03-06
MN428525.1	Mato Grosso	Brazil	2018-03-06
MN428526.1	Mato Grosso	Brazil	2018-03-06
MN428527.1	Mato Grosso	Brazil	2018-03-06
MN428518.1	Mato Grosso	Brazil	2018-03-08
MN428522.1	Mato Grosso	Brazil	2018-03-08
MN428523.1	Mato Grosso	Brazil	2018-03-08
MN428520.1	Mato Grosso	Brazil	2018-03-09
MK156063.1	Bahia	Brazil	2018-03-09
MK156064.1	Bahia	Brazil	2018-03-09
MN428512.1	Mato Grosso	Brazil	2018-03-13
MN428519.1	Mato Grosso	Brazil	2018-03-16
MN428521.1	Mato Grosso	Brazil	2018-03-16
MN428517.1	Mato Grosso	Brazil	2018-03-17
MN428515.1	Mato Grosso	Brazil	2018-03-20
MN428516.1	Mato Grosso	Brazil	2018-03-23
MK244644.1	Rio de Janeiro	Brazil	2018-03-26
MK244647.1	Rio de Janeiro	Brazil	2018-03-27
MN428511.1	Mato Grosso	Brazil	2018-04-18
MN428510.1	Mato Grosso	Brazil	2018-04-25
MN428513.1	Mato Grosso	Brazil	2018-04-26
MN428508.1	Mato Grosso	Brazil	2018-04-27
MN428505.1	Mato Grosso	Brazil	2018-05-21
MT038399.1	Pedro Juan Caballero	Paraguay	2018-06-25
MT038401.1	Pedro Juan Caballero	Paraguay	2018-06-25
MT038402.1	Bella Vista	Paraguay	2018-07-03
MN428507.1	Mato Grosso	Brazil	2018-07-05
MT038403.1	Bella Vista	Paraguay	2018-07-10
MK156058.1	Bahia	Brazil	2018-07-30
MK156062.1	Bahia	Brazil	2018-08-20
MK156055.1	Bahia	Brazil	2018-08-21
MK156054.1	Bahia	Brazil	2018-08-24
MK156061.1	Bahia	Brazil	2018-08-29
MT933029.1	Rio de Janeiro	Brazil	2019-01-21
MT933030.1	Rio de Janeiro	Brazil	2019-01-28

Acession Number	State	Country	Collection date
MT933032.1	Rio de Janeiro	Brazil	2019-02-05
MT933033.1	Rio de Janeiro	Brazil	2019-03-08
MW260512.1	Rio Grande do Norte	Brazil	2019-03-23
MW260513.1	Rio Grande do Norte	Brazil	2019-03-23
MW260515.1	Rio Grande do Norte	Brazil	2019-03-27
MW260514.1	Rio Grande do Norte	Brazil	2019-03-28
MW260516.1	Rio Grande do Norte	Brazil	2019-03-28
MW260517.1	Rio Grande do Norte	Brazil	2019-03-29
MW260518.1	Rio Grande do Norte	Brazil	2019-03-30
MW260519.1	Rio Grande do Norte	Brazil	2019-03-30
MT933035.1	Rio de Janeiro	Brazil	2019-04-17
MT933036.1	Rio de Janeiro	Brazil	2019-04-17
MT933038.1	Rio de Janeiro	Brazil	2019-04-22
MT933039.1	Rio de Janeiro	Brazil	2019-05-06
MT933041.1	Rio de Janeiro	Brazil	2019-05-08
MT933051.1	Rio de Janeiro	Brazil	2019-05-28
MT933043.1	Rio de Janeiro	Brazil	2019-06-07
MT933044.1	Rio de Janeiro	Brazil	2019-06-13
MT933046.1	Rio de Janeiro	Brazil	2019-07-01
MT933049.1	Rio de Janeiro	Brazil	2019-07-07
OL898663.1	São Paulo	Brazil	2020-09-05
OL898714.1	São Paulo	Brazil	2020-11-20
OL898669.1	São Paulo	Brazil	2020-12-17
OL898675.1	São Paulo	Brazil	2020-12-26
OL898682.1	São Paulo	Brazil	2021-01-07
OL898707.1	São Paulo	Brazil	2021-01-07
OL898689.1	São Paulo	Brazil	2021-01-15
OL898690.1	São Paulo	Brazil	2021-01-16
OL898696.1	São Paulo	Brazil	2021-01-28
OL898704.1	São Paulo	Brazil	2021-01-28
OL898664.1	São Paulo	Brazil	2021-01-31
OL898667.1	São Paulo	Brazil	2021-02-01
OL898670.1	São Paulo	Brazil	2021-02-02
OL898676.1	São Paulo	Brazil	2021-02-02
OL898710.1	São Paulo	Brazil	2021-02-02
OL898711.1	São Paulo	Brazil	2021-02-08
OL898697.1	São Paulo	Brazil	2021-02-09
OL898699.1	São Paulo	Brazil	2021-02-09
OL898706.1	São Paulo	Brazil	2021-02-09
OL898677.1	São Paulo	Brazil	2021-02-13
OL898693.1	São Paulo	Brazil	2021-02-13
OL898705.1	São Paulo	Brazil	2021-02-13
OL898684.1	São Paulo	Brazil	2021-02-14
OL898671.1	São Paulo	Brazil	2021-02-16
OL898712.1	São Paulo	Brazil	2021-02-18
OL898681.1	São Paulo	Brazil	2021-02-20
OL898691.1	São Paulo	Brazil	2021-02-21

Acession Number	State	Country	Collection date
OL898665.1	São Paulo	Brazil	2021-02-22
OL898685.1	São Paulo	Brazil	2021-02-22
OL898666.1	São Paulo	Brazil	2021-02-25
OL898672.1	São Paulo	Brazil	2021-02-25
OL898698.1	São Paulo	Brazil	2021-02-25
OL898678.1	São Paulo	Brazil	2021-03-05
OL898692.1	São Paulo	Brazil	2021-03-09
OL898673.1	São Paulo	Brazil	2021-03-15
OL898688.1	São Paulo	Brazil	2021-03-23
OL898686.1	São Paulo	Brazil	2021-03-25
OL898695.1	São Paulo	Brazil	2021-04-04
OL898703.1	São Paulo	Brazil	2021-04-06
OL898679.1	São Paulo	Brazil	2021-04-13
OL898687.1	São Paulo	Brazil	2021-04-16
OL898702.1	São Paulo	Brazil	2021-04-27
OL898713.1	São Paulo	Brazil	2021-04-29
OL898701.1	São Paulo	Brazil	2021-05-05
OL898708.1	São Paulo	Brazil	2021-05-05
OL898715.1	São Paulo	Brazil	2021-05-06
OL898694.1	São Paulo	Brazil	2021-05-11
OL898709.1	São Paulo	Brazil	2021-05-12
TO-UFT-245	Tocantins	Brazil	2021-09-14
TO-UFT-7124	Tocantins	Brazil	2021-07-30
TO-UFT-252	Tocantins	Brazil	2021-10-08
TO-UFT-5070	Tocantins	Brazil	2021-11-18
TO-UFT-22529	Tocantins	Brazil	2022-01-21
TO-UFT-33522	Tocantins	Brazil	2022-01-22
TO-UFT-67522	Tocantins	Brazil	2022-01-24
TO-UFT-5531	Tocantins	Brazil	2022-01-25
TO-UFT-18531	Tocantins	Brazil	2022-01-26
TO-UFT-32531	Tocantins	Brazil	2022-01-26
TO-UFT-50531	Tocantins	Brazil	2022-01-31
TO-UFT-52531	Tocantins	Brazil	2022-01-31
TO-UFT-72569	Tocantins	Brazil	2022-02-07
TO-UFT-86569	Tocantins	Brazil	2022-02-07
TO-UFT-64569	Tocantins	Brazil	2022-02-09
TO-UFT-9217	Tocantins	Brazil	2022-05-27
TO-UFT-9317	Tocantins	Brazil	2022-05-26
TO-UFT-2017	Tocantins	Brazil	2022-05-29
TO-UFT-4345	Tocantins	Brazil	2022-06-21
TO-UFT-8545	Tocantins	Brazil	2022-06-22
TO-UFT-3045	Tocantins	Brazil	2022-06-24
TO-UFT-6145	Tocantins	Brazil	2022-06-24
TO-UFT-4945	Tocantins	Brazil	2022-06-26
TO-UFT-2447	Tocantins	Brazil	2022-07-14
TO-UFT-2645	Tocantins	Brazil	2022-07-20
TO-UFT-6747	Tocantins	Brazil	2022-07-23

Acession Number	State	Country	Collection date
TO-UFT-447	Tocantins	Brazil	2022-07-24

Table S3. Synonymous and missense mutations found in the 27 new CHIKV genomes from Tocantins state.

POS	REF	ALT	Effect	TO-UFT-18531	TO-UFT-2017	TO-UFT-22529	TO-UFT-2447	TO-UFT-245	TO-UFT-252	TO-UFT-2645	TO-UFT-3045	TO-UFT-32531	TO-UFT-33522	TO-UFT-4345	TO-UFT-447	TO-UFT-4945	TO-UFT-50531	TO-UFT-5070	TO-UFT-52531	TO-UFT-5531	TO-UFT-6146	TO-UFT-64569	TO-UFT-6747	TO-UFT-67522	TO-UFT-7124	TO-UFT-72569	TO-UFT-8545	TO-UFT-86569	TO-UFT-9217	TO-UFT-9317
522	A	G	missense	0	0	0	0	1	1	0	0	0	1	0	0	0	0	1	0	1	0	1	0	0	1	0	0	1	0	0
741	C	A	synonymous	0	0	0	0	0	0	0	0	0	0	0	0	0	0	0	0	0	1	0	0	0	0	0	0	0	0	0
844	A	G	missense	0	0	0	1	0	0	0	0	0	0	1	0	0	0	0	0	0	0	0	0	0	0	0	0	0	0	0
908	T	C	synonymous	1	1	1	1	1	1	1	1	1	1	1	1	0	1	1	1	1	1	1	1	1	1	1	1	1	1	1
1202	T	C	synonymous	0	0	0	0	0	0	0	0	0	0	0	0	0	0	0	0	0	0	0	1	0	0	0	0	0	0	0
1204	C	T	missense	0	0	0	0	0	0	1	0	0	0	0	0	0	0	0	0	0	0	0	0	0	0	0	0	0	0	0
1535	C	T	synonymous	0	0	0	0	0	0	0	0	0	0	0	0	0	0	0	0	0	0	0	0	0	0	0	0	1	0	0
1676	G	A	synonymous	0	1	0	0	1	1	1	1	1	1	0	1	1	0	1	1	1	1	1	0	1	1	1	1	1	1	1
1913	C	T	synonymous	0	0	0	1	0	0	1	1	1	1	1	0	1	1	0	1	1	1	1	1	0	0	1	1	1	0	0
1919	T	C	synonymous	0	0	0	1	0	0	1	0	1	1	1	0	1	1	0	1	1	1	1	1	0	0	1	1	1	0	0
2108	T	C	synonymous	0	0	0	0	0	0	1	0	0	0	0	0	0	1	0	1	0	0	0	0	0	0	1	1	0	0	0
2136	C	T	synonymous	0	0	0	0	0	0	0	0	0	0	0	0	0	0	1	0	0	0	0	0	0	0	0	0	0	0	0
2180	T	C	synonymous	0	0	0	0	1	1	1	0	1	1	0	0	0	1	1	1	1	0	1	0	0	1	1	1	1	0	0
2204	C	T	synonymous	1	0	0	0	1	1	1	0	1	1	0	0	0	1	1	1	1	0	1	0	0	1	1	1	1	0	0
2252	T	C	synonymous	1	0	0	1	1	1	1	1	1	1	1	0	1	1	1	1	1	0	1	1	0	1	1	1	1	1	0
2327	A	G	synonymous	1	1	1	1	1	1	1	1	1	1	1	1	1	1	1	1	1	1	1	1	1	1	1	1	1	1	1
2387	C	T	synonymous	0	0	0	0	0	0	0	0	0	0	0	0	0	0	0	0	1	0	0	0	0	0	0	0	1	0	0
2474	T	C	synonymous	1	1	1	1	1	1	1	1	1	1	1	1	1	1	0	1	0	1	1	1	1	1	1	1	1	1	1
2642	T	C	synonymous	0	0	0	0	0	0	0	0	0	0	0	0	0	0	0	0	0	0	0	0	0	0	0	0	0	1	0
2654	G	A	synonymous	1	1	1	1	1	1	1	1	1	1	1	1	1	1	1	1	1	1	1	1	1	1	1	1	1	1	1
2717	A	G	synonymous	0	0	0	0	0	0	0	0	0	0	0	0	0	0	0	0	1	0	0	0	0	0	0	0	1	0	0
2736	C	G	missense	1	1	1	1	1	1	1	1	1	1	1	1	1	1	1	1	1	1	1	1	1	1	1	1	1	1	1
2912	C	T	synonymous	0	0	0	0	0	0	0	0	0	0	0	0	0	0	0	0	0	0	0	0	0	0	0	0	0	0	1
2927	A	G	synonymous	1	1	0	1	1	1	1	1	1	1	1	1	1	1	1	1	0	1	1	1	1	1	1	1	0	1	1

POS	REF	ALT	Effect	TO-UFT-18531	TO-UFT-2017	TO-UFT-22529	TO-UFT-2447	TO-UFT-245	TO-UFT-252	TO-UFT-2645	TO-UFT-3045	TO-UFT-32531	TO-UFT-33522	TO-UFT-4345	TO-UFT-447	TO-UFT-4945	TO-UFT-50531	TO-UFT-5070	TO-UFT-52531	TO-UFT-5531	TO-UFT-6146	TO-UFT-64569	TO-UFT-6747	TO-UFT-67522	TO-UFT-7124	TO-UFT-72569	TO-UFT-8545	TO-UFT-86569	TO-UFT-9217	TO-UFT-9317
3078	A	C	missense	1	1	0	1	1	1	1	1	1	1	1	1	1	1	1	1	1	1	1	1	1	1	1	1	1	1	1
3091	C	T	missense	0	0	0	0	0	0	0	0	0	0	0	0	1	0	0	0	1	0	0	0	0	0	0	0	0	0	0
3095	C	T	synonymous	0	0	0	0	0	0	0	0	0	0	0	0	0	0	0	0	0	1	0	0	0	0	0	0	0	0	0
3300	G	A	missense	0	0	0	0	0	0	0	0	0	0	0	0	0	1	0	1	0	0	0	0	0	0	0	0	0	0	0
3315	G	T	missense	1	1	1	1	1	1	1	1	1	1	1	1	1	1	1	1	1	1	1	1	1	1	1	1	1	1	1
3376	C	T	missense	0	0	1	0	0	0	0	0	0	0	0	0	0	0	0	0	0	0	0	1	0	0	0	0	0	0	1
3440	C	T	synonymous	0	1	0	0	1	1	1	0	0	0	0	0	0	0	1	0	1	1	1	0	0	1	0	1	0	1	0
3446	C	T	synonymous	1	1	1	1	0	0	0	1	1	0	1	1	1	1	0	1	0	1	1	1	0	0	1	0	1	0	1
3463	A	G	missense	0	0	0	0	0	0	0	0	0	0	0	0	0	0	0	0	0	1	0	0	0	0	0	0	0	0	0
3479	C	T	synonymous	0	0	0	0	0	0	0	0	0	0	0	0	0	0	0	0	0	0	0	1	0	0	0	0	0	0	0
3616	T	C	missense	1	0	1	0	0	0	0	0	0	0	0	1	1	1	0	1	0	0	0	0	0	0	1	0	0	0	1
3658	T	C	missense	0	0	0	0	1	0	0	0	0	0	0	0	0	0	0	0	0	0	0	0	0	0	0	0	0	0	0
3863	T	C	synonymous	1	0	1	0	0	0	0	0	0	1	0	0	0	0	0	0	0	0	0	0	1	0	0	0	0	0	0
3932	G	A	synonymous	0	1	0	1	0	0	1	1	0	0	1	1	1	0	0	0	0	1	0	1	0	0	0	1	0	0	1
3974	C	T	synonymous	1	1	1	1	1	1	1	1	1	1	1	1	1	1	1	1	1	1	1	1	1	1	1	1	1	1	1
3986	C	T	synonymous	1	1	1	1	0	0	1	1	1	1	1	1	1	1	0	1	1	1	1	1	1	0	1	1	1	1	1
4079	A	G	synonymous	0	0	0	0	0	1	0	0	0	0	0	0	0	0	0	0	0	0	0	0	0	0	0	0	0	0	0
4232	G	A	synonymous	0	0	0	0	0	0	0	0	0	0	0	0	0	0	0	0	1	0	0	0	0	0	0	0	0	0	0
4292	C	T	synonymous	1	1	1	1	1	1	1	1	1	1	1	1	1	1	1	1	1	1	1	1	1	1	1	1	1	1	1
4463	C	T	synonymous	1	1	1	1	0	1	1	1	1	1	1	1	1	1	0	1	0	1	1	1	1	0	1	1	1	1	1
4688	T	A	synonymous	1	1	1	1	0	0	1	1	1	1	1	1	1	1	0	1	0	1	1	1	1	0	1	1	1	1	1
4820	G	A	synonymous	0	0	0	0	0	0	0	0	0	0	0	0	0	0	0	0	0	0	0	1	0	0	0	0	0	0	0
4899	C	A	synonymous	1	1	1	1	1	1	1	1	1	1	1	1	1	1	1	1	1	1	1	1	1	1	1	1	1	1	1
4916	C	T	synonymous	0	0	0	0	1	1	0	0	0	0	0	0	0	0	1	0	1	0	0	0	0	1	0	0	0	0	0
4927	T	C	missense	0	0	0	0	0	0	1	0	0	0	0	1	0	0	0	0	0	0	0	0	0	0	0	0	0	0	0

POS	REF	ALT	Effect	TO-UFT-18531	TO-UFT-2017	TO-UFT-22529	TO-UFT-2447	TO-UFT-245	TO-UFT-252	TO-UFT-2645	TO-UFT-3045	TO-UFT-32531	TO-UFT-33522	TO-UFT-4345	TO-UFT-447	TO-UFT-4945	TO-UFT-50531	TO-UFT-5070	TO-UFT-52531	TO-UFT-5531	TO-UFT-6146	TO-UFT-64569	TO-UFT-6747	TO-UFT-67522	TO-UFT-7124	TO-UFT-72569	TO-UFT-8545	TO-UFT-86569	TO-UFT-9217	TO-UFT-9317
4994	T	C	synonymous	0	0	0	0	0	0	0	0	0	0	0	0	0	0	0	0	0	0	0	0	1	0	0	0	0	0	0
5068	C	A	missense	0	0	0	0	0	0	0	0	0	0	0	0	0	0	1	0	0	0	0	0	0	0	0	0	0	0	0
5081	T	C	synonymous	1	1	1	1	1	1	1	1	1	1	1	1	1	1	1	1	1	1	1	1	0	1	1	1	1	1	1
5165	C	T	synonymous	0	0	0	0	0	0	0	0	0	0	0	0	0	0	0	0	0	1	0	0	0	0	0	0	0	0	0
5238	G	A	missense	0	1	1	1	0	0	1	1	1	1	1	1	1	1	0	1	1	1	1	1	0	0	1	1	1	1	1
5276	G	A	synonymous	0	1	1	1	1	1	1	1	1	1	1	1	1	1	1	1	1	1	1	1	0	1	1	1	1	1	1
5377	T	C	missense	0	1	1	1	1	1	1	1	1	1	1	1	1	1	1	1	1	1	1	1	0	1	1	1	1	1	1
5449	C	T	missense	0	0	0	0	0	0	0	0	0	0	0	0	0	0	0	0	0	0	0	1	0	0	0	0	0	0	1
5465	T	C	synonymous	0	1	0	1	1	1	1	1	1	1	1	1	1	1	1	1	1	1	1	1	0	1	1	1	1	1	1
5489	A	G	synonymous	0	0	0	0	1	0	0	0	0	0	0	0	0	0	0	0	0	0	0	0	0	0	0	0	0	0	0
5582	C	T	synonymous	0	0	0	0	1	0	0	0	0	0	0	0	0	0	0	0	0	0	0	0	0	0	0	0	0	0	0
5646	T	C	synonymous	0	1	0	1	1	1	1	1	1	1	1	1	1	1	1	1	1	1	1	1	0	1	1	1	1	1	1
5714	A	G	synonymous	0	0	0	0	1	1	0	0	0	0	0	0	0	0	1	0	0	0	0	0	0	1	0	0	0	0	0
5782	A	G	missense	0	1	0	0	0	0	0	0	0	0	0	0	0	0	0	0	0	0	0	0	0	0	1	0	0	0	0
5918	T	C	synonymous	0	0	0	0	0	0	0	0	0	0	0	0	0	0	1	0	0	0	0	0	0	0	0	0	0	0	0
5941	C	A	missense	0	0	0	0	0	0	0	0	0	0	0	0	0	0	1	0	0	0	0	0	0	0	0	0	0	0	0
6009	C	T	synonymous	0	0	0	0	1	1	0	0	1	1	0	0	0	0	1	1	1	0	1	0	0	1	0	0	1	0	0
6359	C	T	synonymous	0	0	0	0	0	0	0	0	0	0	0	0	0	0	0	0	0	0	0	1	0	0	0	0	0	0	0
6500	G	A	synonymous	0	0	0	0	1	0	0	0	0	0	0	0	0	0	0	0	0	0	0	0	0	0	0	0	0	0	0
6615	C	T	missense	0	0	0	0	0	0	0	0	0	0	0	0	0	0	0	0	0	0	0	0	0	0	0	0	1	0	0
6734	C	T	synonymous	1	1	1	1	1	1	1	0	1	1	1	0	1	1	1	1	1	1	1	0	1	1	1	0	1	1	0
6905	C	T	synonymous	0	0	0	0	0	0	0	0	1	0	0	0	0	0	0	0	0	0	0	0	0	0	0	0	0	0	0
7108	C	A	missense	1	1	1	1	1	1	1	1	1	1	1	1	1	1	1	1	1	1	1	1	1	1	1	1	1	1	1
7226	C	T	synonymous	0	0	0	0	0	0	0	0	0	0	0	0	0	0	1	0	0	0	0	0	0	0	0	0	0	0	0
7272	C	T	synonymous	0	1	0	1	1	1	0	0	0	0	1	0	0	0	0	0	1	0	0	0	0	1	0	0	0	0	0

POS	REF	ALT	Effect	TO-UFT-18531	TO-UFT-2017	TO-UFT-22529	TO-UFT-2447	TO-UFT-245	TO-UFT-252	TO-UFT-2645	TO-UFT-3045	TO-UFT-32531	TO-UFT-33522	TO-UFT-4345	TO-UFT-447	TO-UFT-4945	TO-UFT-50531	TO-UFT-5070	TO-UFT-52531	TO-UFT-5531	TO-UFT-6146	TO-UFT-64569	TO-UFT-6747	TO-UFT-67522	TO-UFT-7124	TO-UFT-72569	TO-UFT-8545	TO-UFT-86569	TO-UFT-9217	TO-UFT-9317
7286	C	T	synonymous	1	1	1	1	1	1	1	1	1	1	1	1	1	1	1	1	1	1	1	1	1	1	1	1	1	1	1
7481	T	C	synonymous	0	0	0	0	1	1	0	0	0	0	0	0	0	0	1	1	1	0	1	0	0	1	0	0	1	0	0
7684	G	T	synonymous	0	0	0	0	0	0	0	0	0	0	0	0	0	0	0	1	0	0	0	0	0	0	0	0	0	0	0
7757	A	G	missense	0	0	0	0	0	0	0	0	0	0	0	0	0	0	0	0	0	0	1	0	0	0	0	0	0	0	0
7813	C	T	synonymous	1	0	0	1	1	1	0	0	0	0	1	0	0	1	1	1	1	0	1	0	0	1	0	0	1	0	0
7870	C	T	synonymous	1	1	1	1	1	1	1	1	1	1	1	1	1	1	1	1	1	1	1	1	1	1	1	1	1	1	1
8692	C	T	synonymous	0	1	0	1	1	1	1	0	1	1	0	0	1	1	1	1	1	1	1	1	0	1	0	1	1	1	0
8810	C	T	missense	0	0	0	0	1	0	0	0	0	0	0	0	0	0	0	0	0	0	0	0	0	0	0	0	0	0	0
8896	T	C	synonymous	0	0	0	0	0	0	0	0	0	0	0	0	0	0	0	0	0	0	0	1	0	0	0	0	0	0	0
9005	A	T	missense	0	0	0	0	0	0	0	0	0	0	0	0	0	0	0	0	0	0	0	1	0	0	0	0	0	0	0
9019	C	T	synonymous	0	0	0	0	0	0	0	1	0	0	0	0	0	0	0	0	0	0	0	0	0	0	0	0	0	0	0
9080	T	C	synonymous	1	1	1	1	1	1	1	1	1	1	1	1	1	1	1	1	1	1	1	1	1	1	1	1	1	1	1
9114	C	T	missense	0	0	0	0	0	0	0	0	0	0	0	0	1	0	0	0	0	0	0	0	0	0	0	0	0	0	0
9121	T	C	synonymous	0	0	0	0	0	0	0	0	0	0	0	0	0	1	0	1	0	0	0	0	0	0	0	0	0	0	0
9136	G	A	synonymous	0	0	0	0	0	1	0	0	0	0	0	0	0	0	0	0	0	0	0	0	0	0	0	0	0	0	0
9205	A	G	synonymous	1	0	1	1	0	0	1	1	1	1	1	1	1	1	0	1	1	1	1	1	1	0	1	1	1	1	1
9284	CTT	TTC	missense	1	1	0	1	1	0	0	1	0	0	1	1	1	0	0	1	1	1	1	1	0	0	0	1	1	1	1
9286	T	C	synonymous	0	0	0	0	0	0	1	0	0	1	0	0	0	0	0	0	0	0	0	0	0	0	1	0	0	0	0
9367	C	T	synonymous	0	0	0	0	0	0	0	0	0	0	0	0	0	0	0	0	0	0	0	1	0	0	0	0	0	0	0
9589	T	C	synonymous	0	0	0	0	0	0	0	0	0	0	0	0	0	0	0	0	0	0	0	0	0	1	0	0	0	0	0
9712	G	A	missense	0	0	0	1	0	0	0	0	0	0	1	0	0	0	0	0	0	0	0	0	0	0	0	1	0	0	0
9850	G	A	synonymous	1	1	1	1	1	1	1	1	1	1	1	1	1	1	1	1	1	1	1	1	1	1	1	1	1	1	1
9859	T	C	synonymous	0	0	0	0	0	0	0	0	0	0	0	0	0	0	0	0	0	0	0	1	0	0	0	0	0	0	0
9965	A	C	missense	0	0	0	0	0	1	0	0	0	0	0	0	0	0	0	0	0	0	0	0	0	0	0	0	0	0	0
10098	C	T	missense	0	0	0	0	0	0	0	0	0	0	0	0	0	1	0	1	0	0	0	0	0	0	0	0	0	0	0

POS	REF	ALT	Effect	TO-UFT-18531	TO-UFT-2017	TO-UFT-22529	TO-UFT-2447	TO-UFT-245	TO-UFT-252	TO-UFT-2645	TO-UFT-3045	TO-UFT-32531	TO-UFT-33522	TO-UFT-4345	TO-UFT-447	TO-UFT-4945	TO-UFT-50531	TO-UFT-5070	TO-UFT-52531	TO-UFT-5531	TO-UFT-6146	TO-UFT-64569	TO-UFT-6747	TO-UFT-67522	TO-UFT-7124	TO-UFT-72569	TO-UFT-8545	TO-UFT-86569	TO-UFT-9217	TO-UFT-9317
10286	GCT	ACC	missense	1	1	0	1	0	0	1	1	1	1	1	1	0	1	0	1	0	1	0	1	1	0	1	1	0	1	1
10288	T	C	synonymous	0	0	0	0	0	0	0	0	0	0	0	0	0	0	1	0	0	0	0	0	0	0	0	0	0	0	0
10626	A	C	missense	1	1	1	1	1	1	1	1	1	1	1	1	1	1	1	1	1	1	1	1	1	1	1	1	1	1	1
10745	C	T	synonymous	0	0	0	0	0	0	0	1	0	0	0	0	0	0	0	0	0	0	0	0	0	0	0	0	0	0	0
10799	G	A	missense	1	1	1	1	1	1	1	1	1	1	1	1	1	1	1	1	1	1	1	1	1	1	1	1	1	1	1
10907	G	A	missense	1	1	0	1	1	1	1	1	1	1	1	1	1	1	1	1	1	1	1	1	1	1	1	1	1	1	1
10930	C	T	synonymous	0	1	0	0	0	0	1	1	0	0	0	0	0	0	0	0	0	0	0	1	0	0	0	1	0	0	1
11008	A	T	synonymous	1	1	0	1	0	0	1	1	1	1	1	1	0	1	0	1	0	1	1	1	1	0	1	1	1	1	1
11091	G	T	missense	0	0	0	0	0	0	0	0	0	0	0	0	0	0	0	0	0	0	0	1	0	0	0	0	0	0	1
11257	C	T	synonymous	0	0	0	0	1	0	0	0	0	0	0	0	0	0	0	0	0	0	0	0	0	0	0	0	0	0	0
11302	C	T	synonymous	0	0	0	0	1	1	0	0	0	0	0	0	0	0	1	0	0	0	0	0	0	1	0	0	0	0	0
